# Body image and risk of exercise addiction in adults: A systematic review and meta-analysis

**DOI:** 10.1556/2006.2024.00085

**Published:** 2025-02-06

**Authors:** Shuai Guo, Agata Kamionka, Qinbo Xue, Bernadetta Izydorczyk, Małgorzata Lipowska, Mariusz Lipowski

**Affiliations:** 1 Faculty of Sport and Leisure, Guangdong Ocean University, Zhanjiang, China; 2 College of Physical Education, Yangzhou University, Yangzhou, China; 3 Department of Psychology, Gdansk University of Physical Education and Sport, Gdansk, Poland; 4 Institute of Psychology, Jagiellonian University, Krakow, Poland; 5 Institute of Psychology, University of Gdansk, Gdansk, Poland; 6 Faculty of Social and Humanities, WSB University in Gdansk, Gdansk, Poland

**Keywords:** body image, exercise addiction, meta-analysis, cognitive component, adults

## Abstract

**Background and aims:**

Exercise addiction in adults is increasingly recognized as a public health concern, carrying potentially harmful physical, psychological, and social consequences. Body image—encompassing cognitive, behavioral, affective, and perceptual elements—may be a key factor in this risk, yet comprehensive evidence remains scarce. This systematic review and meta-analysis aimed to (1) evaluate the overall relationship between multidimensional body image and the risk of exercise addiction in adults, and (2) identify key moderators (e.g., different elements of body image, gender, region).

**Methods:**

A systematic search was conducted in PsycINFO, Medline, SPORT Discus, Web of Science, and Embase up to October 22, 2024. A three-level meta-analysis model was employed, and univariate and multivariate meta-regressions were used to explore potential moderating factors.

**Results:**

A total of 38 eligible studies were identified, contributing 65 effect sizes. The meta-analysis revealed a moderate correlation (r = 0.26) between body image and risk of exercise addiction in adults. Moreover, the cognitive component of body image and the use of compulsive exercise measures in addiction assessments emerged as unique moderators, each further strengthening the observed relationship.

**Discussion and conclusions:**

These findings underscore the central role of body image—particularly its cognitive dimension—in shaping the risk of exercise addiction in adults, especially when assessed via compulsive exercise measures. Incorporating these insights into research and practice may guide more effective interventions and improve public health strategies aimed at mitigating harmful exercise behaviors.

## Introduction

Exercise addiction is a dysfunctional behavior characterized by excessive exercise, loss of control over exercise behavior, and possible negative consequences on physical, psychological, or social well-being (Juwono & Szabo, 2021; Weinstein & Szabo, 2023). Although regular exercise is considered an effective strategy for human health promotion (Thompson et al., 2020), evidence suggests that some individuals may develop potentially dysfunctional patterns of exercise behavior (Marques et al., 2019). This is a complex phenomenon, which implies that exercisers lose control over their exercise behavior to the extent that they experience negative physiological and social symptoms, such as physical injury or immune problems, withdrawal symptoms, impairment or loss of social relationships (Juwono & Szabo, 2021; Szabo, Demetrovics, & Griffiths, 2018). In the past five decades of research, scholars have used different terms to study this phenomenon, including compulsive exercise, obligatory exercise, exercise dependence, exercise addiction, and others (Lichtenstein, Hinze, Emborg, Thomsen, & Hemmingsen, 2017). Exercise addiction is considered by some scholars to be the most appropriate term because it encompasses both dependence and compulsion characteristics (Szabo, Griffiths, de La Vega Marcos, Mervó, & Demetrovics, 2015; Weinstein & Szabo, 2023). Therefore, throughout this review, the term exercise addiction will be used to refer to this phenomenon.

The most recent systematic reviews on the prevalence of exercise addiction revealed that although the highest prevalence (6%–9%) was found among athletes, the prevalence among the general population (3%–8%) was also notable, such as 5.5% in university students and 8.1% in regular exercisers (Marques et al., 2019; Trott et al., 2020). The risk of exercise addiction was widely reported in studies from different countries and regions, such as Europe, America, and Asia, and is becoming a global public health issue that needs to be addressed (Marques et al., 2019; Reynolds, Plateau, & Haycraft, 2022). In light of this, identifying potential risk factors of exercise addiction were an important research area.

In sport and exercise psychology research, body image has been considered an important variable related to physical activity and exercise behavior of people (Sabiston, Pila, Vani, & Thogersen-Ntoumani, 2019). According to the theoretical models for exercise addiction, exercise may become an addiction through positive or negative reinforcement processes related to body image (Egorov & Szabo, 2013). In psychological research, body image is conceptualized as a subjective mental schema of individuals concerning body appearance, attractiveness, health and function (Cash, 2004; Prnjak, Jukic, Mitchison, Griffiths, & Hay, 2022). It is a multidimensional construct which includes cognition (e.g., body dissatisfaction), behavior (e.g., body checking and avoidance), affect (e.g., body anxiety, shame) and perception (e.g., body visual perception) (Prnjak et al., 2022; Thompson & Schaefer, 2019). Empirical evidence suggests that body image dissatisfaction is a common risk factor for the development of exercise addiction (Alcaraz-Ibáñez, Paterna, Sicilia, & Griffiths, 2021; Zou et al., 2022); body function-related shame and pride are positively linked to exercise addiction (Alcaraz-Ibáñez, Sicilia, Dumitru, Paterna, & Griffiths, 2019). The body image of multidimensional construct may help explain the development and maintenance of exercise addiction. However, most current studies only examine the relationship between one or a category of elements of body image and risk of exercise addiction, neglecting the holistic assessment of body image. Given the complexity and specificity of the relationship between the body image of multidimensional construct and risk of exercise addiction, it is imperative to systematically investigate the relationship between body image and risk of exercise addiction.

Although the relationship between various aspects of body image and exercise addiction has been investigated for over 20 years, no systematic and comprehensive review has yet synthesized the existing evidence on the relationship between the body image of multidimensional construct and risk of exercise addiction. Previous studies have used different terms (e.g., compulsive exercise, maladaptive exercise, morbid exercise behavior, etc.) to conduct systematic reviews on exercise addiction, covering topics such as its prevalence (Marques et al., 2019), existing research reviews (Weinstein & Szabo, 2023), its association with eating disorders (Trott et al., 2020), and sociocultural influences on it (Reynolds et al., 2022). However, only a few studies have reviewed the relationship between an element of body image and risk of exercise addiction, such as systematically examined the relationship between body dissatisfaction and risk of exercise addiction (Alcaraz-Ibáñez et al., 2021). Given that body image is a multidimensional construct, different dimensions may have distinct associations with risk of exercise addiction. Therefore, we conducted a systematic review to explore the relationship between the body image of multidimensional construct and risk of exercise addiction. Our review focused on adults other than elite athletes (those competing at professional, Olympic, or collegiate/university levels) and sports professionals or practitioners (Rice et al., 2019). This is because the overtraining and overcommitment of elite athletes are not comparable to exercise addiction (Weinstein & Szabo, 2023), and sports professionals or practitioners differ significantly from non-professionals in the prevalence and determinants of exercise addiction (Egorov & Szabo, 2013; Marques et al., 2019). Meanwhile, we also considered the number of published relevant papers and the research fields of the author teams.

The main aim of this systematic review was to examine the current state of the science on the relationship between body image and risk of exercise addiction in adults and elucidate the moderating effects of various factors (e.g., different elements of body image, gender, region) on this relationship. The results of this systematic review may identify key elements in body image that significantly impact the risk of exercise addiction among adults, which could translate into improved professional practice for physical activity and health professionals. Apart from that, the research results might also provide evidence to address adverse impacts associated with physical activity, which maximizes the potential benefits of exercising for health.

## Methods

This review was conducted in accordance with the Preferred Reporting Items for Systematic Reviews and Meta-Analyses (PRISMA) framework (Page et al., 2021) (see Supplementary Material A). The review was registered with PROSPERO (No. CRD42023399118), and the protocol was submitted.

### Search strategy

The main author (SG) conducted the search with the guidance of a librarian experienced in systematic review methods. A systematic search of five databases (PsycINFO, Medline, SPORT Discus, Web of Science, Embase) examined body image and exercise addiction published by October 2024 (see Supplementary Material B). The final search date registered in PROSPERO for this study was February 28, 2022. However, due to the time required to complete the study and the manuscript submission process, the research team, following the reviewers' recommendations, updated the search. The final search was conducted on October 22, 2024. We did not limit the earliest publication date, instead utilizing the full range of publication years available in each database to ensure comprehensive coverage.

The records found were published from 1982 to 2024. Free-text words for body image derived from previous studies were used in all databases (Nowicki, Marchwinski, O’Flynn, Griffths, & Rodgers, 2022; Sabiston et al., 2019). Free-text words for exercise addiction was derived from studies investigating similar outcomes (Alcaraz-Ibáñez, Paterna, Griffiths, Demetrovics, & Sicilia, 2022; Marques et al., 2019; Martenstyn, Touyz, & Maguire, 2021). Search terms included two sets of terms: (1) body image OR ( (awareness or affect or appreciat* or areas satisfaction or assessment or behav* or critici* or cognition* or concern* or discrepancy or dissatisfaction or distortion or disturbance or drive for muscularity or embarrass* or envy or envious or experience? or function* or guilt or perception? or pride or proud or project* or representation* or questionnaire? or schema* or shame* or satisfaction or scale?) and (appearance or body) ) OR ( (concern* or internalization* or embarrassment or emotion* or envy or envious or guilt or pride or shame) and weight ) OR ( ((objectification OR physical) AND appearance OR physical) AND attractiveness ) OR body image OR ( (embarrassment OR envy OR shame OR pride OR guilt*) AND 'body related' ); (2) compulsive exercise OR ( obligatory exercise* or exercise dependence or exercise addiction or compulsive exercise or sport dependence or sport addiction or obligatory sport or physical activity dependence or physical activity addiction or obligatory physical activity or physical activity compulsive or run* dependence or run* addiction or obligatory run* or compulsive run* or bigorexia or excessive exercise* or unhealth exercise* or exercise abuse or exercise pathology or pathological exercise or overexercise* or over-exercise* or compulsive exercising or compulsive sporting or sports addiction). We did not set age terms during the search but considered age during the screening process. Database searches were limited to peer-reviewed journal articles in English. Manual searches of systematic reviews on exercise addiction and the references of the eligible articles were performed to identify additional eligible articles.

### Study selection

The search results were imported into the EndNote X9 software and, after duplicate records were removed, the study selection was performed using the Covidence software. In the first stage, SG and QX performed title and abstract screening and discarded articles deemed irrelevant. The inter-rater agreement was high, with a Cohen's kappa values of 0.76 (99% agreement). We defined relevance as studies that describing the relationship between body image and risk of exercise addiction. All disagreements were resolved through discussions with the corresponding authors. In the second phase, the full texts of potential studies for inclusion in the review were reviewed by SG, AK, and QX and screened according to the inclusion and exclusion criteria in Table 1. In this process, the inter-rater reliability for Cohen's Kappa was 0.89 (94% agreement). Disagreements on the full-text review were resolved through discussions with the corresponding authors. The corresponding author assessed the final set of included studies to confirm the inclusion and exclusion criteria were met.

**Table 1.** Inclusion and Exclusion Criteria of this study

Inclusion criteria	Exclusion criteria
Studies targeting only adult participants (ages between 18 and 64 years) (Bull et al., 2020).	Studies targeting clinical or treatment-seeking populations (e.g., cardiovascular disease, pregnancy, mental illness). If the sample was in a medical weight loss program, overweight and obesity would be considered clinical.
Relevance defined as studies describing the relationship between body image and risk of exercise addiction.	Studies not reporting numerical data on the relationship between body image and risk of exercise addiction.
Studies reporting precise body image measurements.	Studies targeting elite athletes, sports professionals, or persons with disabilities (including para-athletes, professional youth, and Olympic athletes) (Rice et al., 2019; Swann, Moran, & Piggott, 2015).
Studies with explicit exercise addiction measurements.	Studies without precise body image measurements (e.g., focused only on objective or self-reported weight).
limited to peer-reviewed journal articles in English	Studies without explicit exercise addiction measurements.

### Data extraction

A coding framework (see Supplementary Material C) was developed based on common characteristics of preliminarily identified studies and was piloted. Referring to body image (Dahlenburg, Gleaves, Hutchinson, & Coro, 2020; Thompson, Burke, & Krawczyk, 2012) and exercise addiction (Alcaraz-Ibáñez, Paterna, Sicilia, & Griffiths, 2022; Çakın, Juwono, Potenza, & Szabo, 2021) studies, the included studies are grouped according to the measurement tools of body image and risk of exercise addiction (see Supplementary Material D). Using this resulting coding sheet, SG and QX independently extracted pertinent data from the qualifying studies. The inter-rater agreement, measured by Cohen's kappa, ranged from 0.75 to 0.94 (88–98% agreement), which indicates good reliability. Disagreements and issues among authors were resolved through discussion with the participation of the corresponding author.

### Quality assessment

This study has conducted a quality assessment of the included studies using the Quality of Survey Studies in Psychology (Q-SSP) checklist (Protogerou & Hagger, 2020). To better adapt to the background of this study, we have made the following modifications to the Q-SSP checklist, referring to the published systematic reviews (Parry et al., 2021). (1) For item 1 (reporting of hypothesis or research question), we have accepted stating a purpose or objective without reporting a research question or hypothesis as qualified. (2) For studies that collected data through online platforms, items 13 (information on personnel who collected data) and 14 (information on the context of collection data) were coded as not applicable. (3) For item 19 (reporting to participants at the end), considering that many studies did not have the conditions to report after data collection (such as online surveys), we have coded this item as not applicable. With these revisions in mind, the checklist we used may include 17 or 19 items (the original checklist had 20). Based on the Q-SSP study (Protogerou & Hagger, 2020), scores of greater than or equal to 70 are considered acceptable quality and scores of less than 70 are considered questionable quality. SG and AK have assessed each study independently using the Q-SSP checklist, and all disagreements were resolved through discussion with the involvement of the corresponding author. Ultimately, all but one of the studies have scored above 70 (see Table 2; Supplementary Material E).

**Table 2.** Characteristics of the studies included in the present study

Study	Region	Gender/N	Mean age	Population	Mean BMI	Body image measure	Risk of exercise addiction measure	*K*	Study design	Q-SSP score
Pasman and Thompson (1988)	USA	F/30; M/30	NA	Runners; exercisers in fitness centers	NA	Eating Disorder Inventory – Body Dissatisfaction Subscale; Body Self Relations Questionnaire – Physical Appearance Evaluation Subscale	Obligatory Exercise Questionnaire (OEQ)	8	Cross-sectional	78.95
Diehl, Johnson, Rogers, and Petrie (1998)	USA	F/160	21.53	General university students	22.22	Social Physique Anxiety Scale	Obligatory Exercise Questionnaire (OEQ)	1	Cross-sectional	73.68
Gulker, Laskis, and Kuba (2001)	USA	F&M/172	36.00	Regular exercisers	NA	Eating Disorder Inventory 2 – Body Dissatisfaction Subscale	Obligatory Exercise Questionnaire (OEQ)	1	Cross-sectional	73.68
Mussap (2006)	Australia	M/120	25.94	General university students	24.35	Eating Disorder Inventory 2 – Body Dissatisfaction Subscale	Obligatory Exercise Questionnaire (OEQ)	1	Cross-sectional	73.68
Mussap (2007)	Australia	F/130	25.10	General university students	NA	Eating Disorder Inventory 2 – Body Dissatisfaction Subscale	Obligatory Exercise Questionnaire (OEQ)	1	Cross-sectional	68.42
Chu, Bushman, and Woodard (2008)	USA	F/200; M/137	NA	General university students	NA	Social Physique Anxiety Scale	Obligatory Exercise Questionnaire (OEQ)	2	Cross-sectional	84.21
Bushman and Brandenburg (2009)	USA	F170; M/141	NA	General university students	NA	Social Physique Anxiety Scale	Obligatory Exercise Questionnaire (OEQ)	2	Cross-sectional	89.47
Fortier and Farrell (2009)	Canada	F&M/110	NA	Regular exercisers	NA	Body Cathexis Scale	Commitment to Exercise Scale (CES)	1	Cross-sectional	84.21
Chittester and Hausenblas (2009)	USA	M/113	20.34	General university students	25.05	Drive for Muscularity Scale – Muscle-oriented body image subscale/Muscularity-related behavior subscale	Exercise Dependence Scale (EDS)	2	Cross-sectional	94.74
De Young and Anderson (2010)	USA	F&M/226	19.30	General university students	NA	Eating Disorder Examination Questionnaire – Shape Concern Subscales/Weight Concern Subscales	Obligatory Exercise Questionnaire (OEQ)	2	Cross-sectional	73.68
Boroughs, Krawczyk, and Thompson (2010)	USA	F&M/1041	20.95	General university students	NA	Multidimensional Body Self-Relations Questionnaire – Appearance Evaluation Scale/Body Areas Satisfaction Scale	Obligatory Exercise Questionnaire (OEQ)	2	Cross-sectional	76.47
Homan (2010)	USA	F/231	19.20	General university students	22.00	Multidimensional Body-Self Relations Questionnaire – Body Areas Satisfaction Subscales	Obligatory Exercise Questionnaire (OEQ)	1	Longitudinal	89.47
Gapin and Petruzzello (2011)	USA	F&M/179	35.88	Runners	22.71	Eating Disorder Inventory – Body Dissatisfaction Scale	Obligatory Exercise Questionnaire (OEQ)	1	Cross-sectional	88.24
Lamarche and Gammage (2012)	Canada	F/231	22.92	General university students	22.98	Appearance Schemas Inventory-Revised – self-evaluative salience Subscale/motivational salience Subscale; Body-image Ideals Questionnaire – discrepancy subscale	Exercise Dependence Scale (EDS)	3	Cross-sectional	84.21
LePage, Price, O’Neil, and Crowther (2012)	USA	F/51; F/76	19.06; 19.08	Regular exercisers	NA	Body Shape Questionnaire	Obligatory Exercise Questionnaire (OEQ)	2	Longitudinal	84.21
Cook et al. (2015)	USA	F/1766	36.98	Runners	23.78	Social Physique Anxiety Scale	Exercise Dependence Scale (EDS)	1	Cross-sectional	94.12
Bell, Donovan, and Ramme (2016)	Australia	F/388	21.46	General university students	23.00	Body Image and Body Change Questionnaire – Body Image Concern subscale	Obligatory Exercise Questionnaire (OEQ)	1	Cross-sectional	76.47
Brewster, Sandil, DeBlaere, Breslow, and Eklund (2017)	USA	M/326	28.71	General public	NA	Objectified Body Consciousness Scale – body surveillance subscale; Body Parts Satisfaction Scale for Men; Drive for Muscularity Scale	Compulsive Exercise Test (CET)	3	Cross-sectional	82.35
Martin and Racine (2017)	USA	F&M/531	19.37	General university students	24.00	Eating Pathology Symptoms Inventory – Body Dissatisfaction subscale	Commitment to Exercise Scale (CES)	1	Cross-sectional	76.47
Patterson and Goodson (2017)	USA	F/206	NA	General university students	NA	single-factor Body Shape Questionnaire	Compulsive Exercise Test (CET)	1	Cross-sectional	84.21
Ertl et al. (2018)	USA	F/322	19.89	General university students	NA	Objectified Body Consciousness Scale – Body Shame Subscale	Exercise Addiction Inventory-Short Form (EAI)	1	Cross-sectional	76.47
Uhlmann, Donovan, Zimmer-Gembeck, Bell, and Ramme (2018)	Australia	F/356	20.57	General university students	22.79	Body Image and Body Change Inventory – Body Image Concern subscale	Obligatory Exercise Questionnaire (OEQ)	1	Cross-sectional	70.59
Liu, Chang, Gill, Wu, and Lu (2018)	Taiwan, China	M/278	29.03	Exercisers in fitness centers	22.57	Muscular Figure Rating Scale; Drive for Muscularity Scale	Exercise Dependence Scale-Revised (EDS-R)	2	Cross-sectional	89.47
Donovan, Uhlmann, and Loxton (2020)	Australia	F/558	22.06	General university students	23.57	Fit-Ideal Internalization Test; Body-Image Ideals Questionnaire	Compulsive Exercise Test (CET)	2	Cross-sectional	70.59
Kelly, Kosty, Guerricabeitia, Guidinger, and Williamson (2020)	USA	M/1109	24.10	General public	25.40	Revised Male Body Image Attitudes Scale – body fat subscale/muscularity subscale	Exercise Dependence Scale (EDS)	2	Cross-sectional	94.12
Dawson and Hammer (2020)	USA	M/632	28.31	General public	NA	Male Body Attitudes Scale – Body Fat Dissatisfaction/Muscle Dissatisfaction	Exercise Dependence Scale-Revised (EDS-R)	2	Cross-sectional	82.35
Prochnow, Patterson, and Umstattd Meyer (2021)	USA	F/208	19.40	General university students	22.10	single-factor Body Shape Questionnaire	Compulsive Exercise Test (CET)	1	Cross-sectional	84.21
Back, Josefsson, Ivarsson, and Gustafsson (2021)	Sweden	F&M/330	30.10	Exercisers in university exercise group	NA	Multidimensional Body-Self Relations Questionnaire – Appearance Orientation Subscale	Exercise Dependence Scale-Revised (EDS-R)	1	Cross-sectional	78.95
Gori, Topino, and Griffiths (2021)	Italy	F&M/319	30.78	Regular exercisers	NA	Eating Disorder Inventory-3-Referral Form – body dissatisfaction subscale; Body Image Concern Inventory	Exercise Addiction Inventory (EAI)	2	Cross-sectional	76.47
Gori, Topino, Pucci, and Griffiths (2021)	Italy	F&M/288	28.35	Regular exercisers	NA	Body Image Concern Inventory – Dysmorphic Symptoms Subscale/Symptom Interference Subscale	Exercise Addiction Inventory (EAI)	2	Cross-sectional	70.59
Bonfanti, Lo Coco, Salerno, and Di Blasi (2022)	Italy	F&M/194	25.91	Exercisers in fitness centers	22.23	Drive for Leanness Scale	Exercise Addiction Inventory (EAI)	1	Cross-sectional	82.35
Donovan and Uhlmann (2022)	Australia	F/448	18.83	General university students	23.05	Fit Ideal Internalization Test; Objectified Body Consciousness Scale – Body Surveillance Subscale; Body-Image Ideals Questionnaire	Compulsive Exercise Test (CET)	3	Cross-sectional	70.59
Novita, Andriani, Erika Lipowski, and Lipowska (2022)	Poland; Indonesia	F&M/709; F&M/172	32.39; 23.77	General university students	NA	Fear of Negative Appearance Evaluation Scale	Obligatory Exercise Questionnaire (OEQ)	2	Cross-sectional	76.47
Mader, Müller, Wölfling, Beutel, and Scherer (2023)	Germany	F&M/122	25.85	General public	NA	Eating Disorder Examination Questionnaire – Shape concern scale/Weight concern scale	Exercise Dependence Scale-21 (EDS-21)	2	Cross-sectional	70.59
Palermo and Rancourt (2023)	USA	F&M/446	20.10	General university students	NA	The Body Uneasiness Test-Body Image Concerns subscale	Exercise and Eating Disorder Questionnaire (EED) – Compulsive and Positive and Healthy Exercise subscales	1	Cross-sectional	88.24
Akbari, Seydavi, Zamani, and Griffiths (2024)	Iran	F&M/745	26.19	General public	23.59	Body Image Concern Inventory	Exercise Addiction Inventory (EAI)	1	Cross-sectional	88.24
Gori, Topino, and Griffiths (2024)	Italy	F&M/300	30.30	Regular exercisers	NA	Body Image Concern Inventory	Exercise Addiction Inventory (EAI)	1	Cross-sectional	76.47
Soraci et al. (2024)	Italy	F&M/235	35.17	General public	NA	Body Esteem Scale	Exercise Addiction Inventory (EAI)	1	Cross-sectional	100

*Note:* N = Number of the samples; F = Female; M = Male; NA = Not available; *K* = Number of effect sizes; Q-SSP score = Quality assessment score of the study.

### Data analysis

All analyses were performed in R version 4.2.2.

1. *Standardized effect sizes.* Most included studies quantified the relationship between body image and risk of exercise addiction using Pearson's *r*. Adhering to the recommendations of the existing study (Ringquist, 2013), we converted the *r* effect sizes to Fisher's *Z* for analysis. For studies that reported results through between-group comparisons, we used the “esc” package to convert to Fisher's *Z*. For studies that reported results using regression tables, we used the online calculator “Psychometrica” (Lenhard & Lenhard, 2016) to convert the standardized beta coefficients to *r* and then to Fisher's *Z*. Prior to the formal meta-analysis, we checked for outlier effect sizes in the dataset by screening for Fisher's *Z* values greater than 3.29 or less than −3.29 (Tabachnick & Fidell, 2013).
2. *Main analysis and heterogeneity tests.* Given the dependence between effect sizes extracted in this study, we conducted a meta-analysis using the three-level random-effects model with REML estimation in the “metafor” package (Assink & Wibbelink, 2016). Some articles included in this meta-analysis reported multiple effect sizes, showing correlations between effect sizes, such as effect sizes of different measurement tools for the same population or effect sizes of different population subgroups for the same sample. This issue is better addressed by a three-level random-effects model that considers three different variance components, including the sampling variance of effect sizes (level 1), the variance between effect sizes within studies (level 2), and the variance between studies (level 3) (Alcaraz-Ibáñez, Paterna, & Griffiths, 2023; Assink & Wibbelink, 2016). An alternative method for handling dependency among effect sizes is robust variance estimation (Hedges, Tipton, & Johnson, 2010). However, the multilevel random effects model manages within-study dependencies by slightly inflating study-level variance, resulting in more reliable standard errors (Van den Noortgate, López-López, Marín-Martínez, & Sánchez-Meca, 2013). This approach is particularly effective when individual studies contribute only a limited number of effect sizes (Rodgers & Pustejovsky, 2020). Additionally, robust variance estimation may encounter challenges when moderators are unevenly distributed across studies (Tanner-Smith, Tipton, & Polanin, 2016). For these reasons, the multilevel model was chosen as the most appropriate method for the current analysis.
  Following similar studies (Khanna et al., 2021), we used restricted maximum likelihood (REML) to estimate parameter models considering the dependency among effect sizes. By applying the adjustment of Knapp and Hartung (Knapp & Hartung, 2003), we used the *t*-distribution to assess the significance of the model coefficients and to establish the corresponding confidence intervals. The effect sizes and their 95% confidence intervals were *z* to *r* transformed for a more straightforward interpretation of the results (Borenstein, Hedges, Higgins, & Rothstein, 2009). The correlated effect sizes were interpreted as very large (≥0.40), large (≥0.30), moderate (≥0.20), small (≥0.10), and very small (≥0.05) when reliably estimated (Funder & Ozer, 2019; Gignac & Szodorai, 2016). Furthermore, we performed two separate log-likelihood-ratio tests to determine whether within-study variance (level 2) and between-study variance were significant and analyzed the distribution of total variance across the three levels (Assink & Wibbelink, 2016; Park & Beretvas, 2019). We constructed a forest plot and examined heterogeneity for all analyses using *Q* tests and *I*^*2*^. A statistically significant *Q* value indicates heterogeneity, and *I*^*2*^ values of 25%, 50%, and 75% were described as low, moderate, and high heterogeneity, respectively (Higgins, Thompson, Deeks, & Altman, 2003). The effect of each effect size on the total effect was examined by influence diagnosis and Leave-one-out sensitivity analysis (for-loop).

3. *Publication bias.* Based on similar studies (Alcaraz-Ibáñez et al., 2023), we employed a likelihood ratio test from a three-parameter selection model (3PSM) to examine publication bias. Methodological research suggests that the likelihood ratio test of 3PSM provides the best estimation and hypothesis testing for evaluating publication bias when there is high heterogeneity and the true effect size is below 0.3 (Carter, Schönbrodt, Gervais, & Hilgard, 2019; McShane, Böckenholt, & Hansen, 2016; Rodgers & Pustejovsky, 2021). We used the “weightr” package to conduct the likelihood ratio test of 3PSM, and a significant statistical result indicates the possibility of publication bias.
4. *Moderation analysis.* In cases with the significant variance within and/or between studies, we estimated moderators of the overall effect by introducing moderating variables in a three-level meta-analytic model. First, we evaluated potential moderating effects separately, including measurement type for body image, different exercise addiction measurement tools, participant population, mean age, mean BMI, study region, gender, publication year, and study design. For categorical variables, we transformed them into *K*-1 dummy variables using binary codes. For continuous variables, we created new variables centered on their mean values. Second, we performed multiple moderator model analysis on all variables identified as significant moderators in the separate moderator effects analysis to examine the unique effects of significant moderators.

## Results

### Study characteristics

**Fig. 1.**
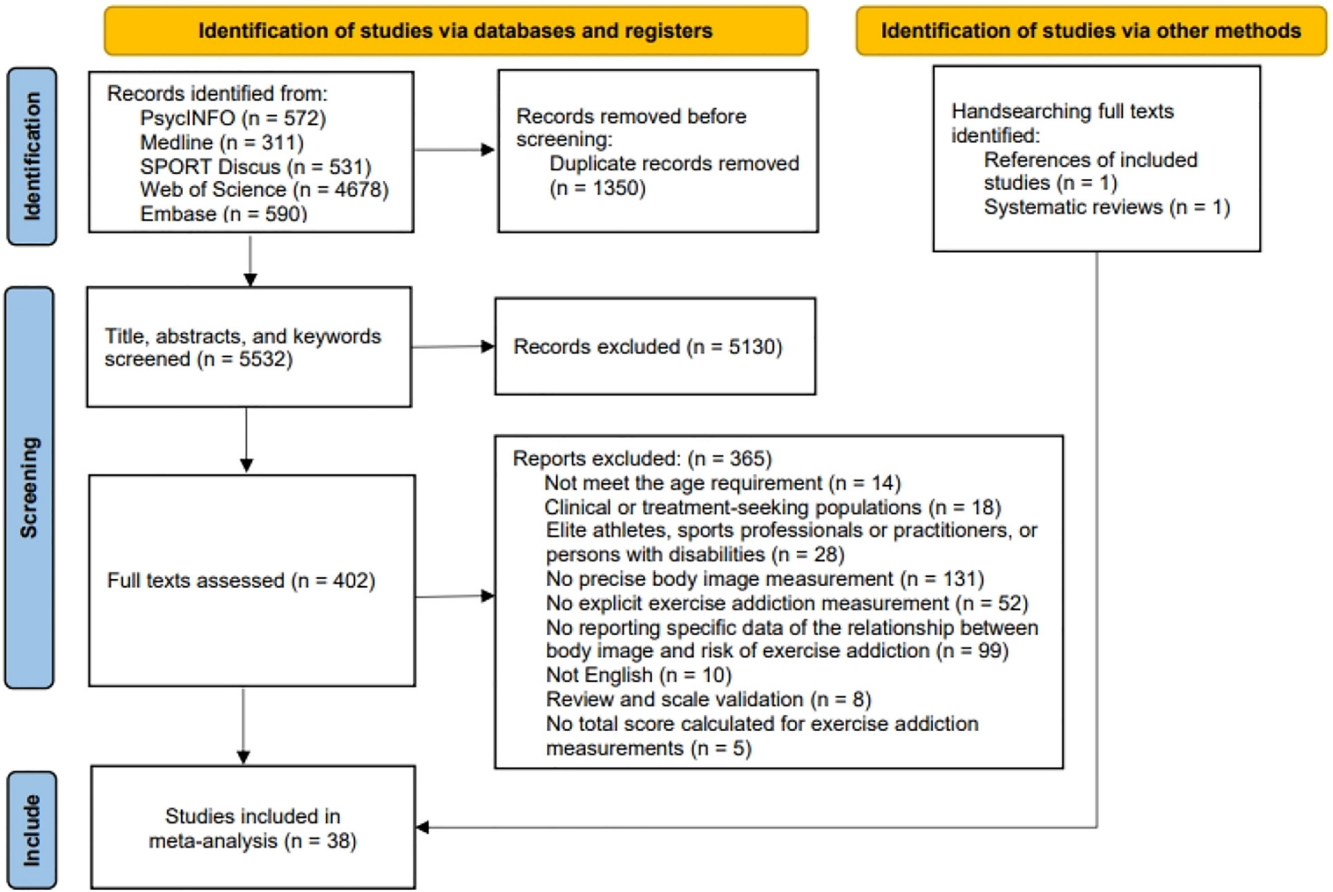
PRISMA flow diagram of study selection

The initial search produced 6,682 results. After eligibility screening (see Fig. 1), 38 studies were included in the final sample. From these studies, 65 effect sizes involving 21,412 participants were included in the analysis (see Table 2; Supplementary Material F). All included studies were publicly published journal articles that underwent peer review, with the earliest study from 1988. All studies used a cross-sectional design except for 2 studies (*K* = 3) that adopted a longitudinal design. These studies covered different countries and regions, with 22 from North America (Canada = 2, USA = 20; *K* = 41), 6 from Australia (*K* = 9), 8 from Europe (Germany = 1, Italy = 5, Poland = 1, Sweden = 1; *K* = 11), and 3 from other countries and regions (Chinese Taipei = 1, Indonesia = 1, Iran = 1; *K* = 4). Notably, one study conducted a cross-cultural comparison between Poland and Indonesia, reporting effect sizes for both countries separately. Participants were primarily categorized into five categories: general university students (*K* = 31), the general public (*K* = 11), regular exercisers (at least 3 times per week; *K* = 9), runners (participants from road race events and running clubs; *K* = 6), and other populations (exercisers in fitness centers and university exercise group; *K* = 8). After setting the unreported mean age and BMI as “NA”, the weighted mean age of the participants was 24.84 (*K* = 51), and the weighted mean BMI was 23.36 (*K* = 25). Of all reported effect sizes, 18 were for males, 25 were for females, and 22 were for mixed-gender samples.

Body image was assessed using different tools (see Supplementary Material D), mainly categorized as global and site-specific satisfaction (dissatisfaction with appearance and weight; *K* = 44), affective measures (negative feelings and emotions towards one's body; *K* = 9), behavioral measures (demonstrating dissatisfaction with body image through personal behavior; *K* = 1), and cognitive measures (thoughts and beliefs about one's appearance; *K* = 11). The evaluation of risk of exercise addiction mainly utilized the obligatory exercise measure (OEQ; *K* = 28), exercise dependence measures (EDS, EDS-R ; *K* = 15), compulsive exercise measures (CET, EED-Compulsive and Positive and Healthy Exercise Subscales; *K* = 11), exercise addiction measures (EAI; *K* = 9), and commitment to exercise measure (CES; *K* = 2), all of which reported the relationship with body image through total scores.

### Correlations between body image and risk of exercise addiction in adults

The current study utilized a three-level random effects model to examine the correlation between body image and risk of exercise addiction in adults. The meta-analysis results indicate a significant and positive overall relationship between the two variables (*r* = 0.26, *p* < 0.001, 95% CI = 0.19 to 0.32; see Supplementary Material G for the forest plot), with a moderate effect size. The *Q*-test (*Q* = 995.96, *p* < 0.001) revealed significant heterogeneity. Two separate tests of the log-likelihood-ratio determined the presence of significant within-study variance (level 2, *p* < 0.001) and between-study variance (level 3, *p* < 0.001). Of the total variance, 6.58% could be attributed to sampling variance (level 1), 26.92% to within-study variance (level 2), and 66.50% to between-study variance (level 3). If the proportion of variance attributable to sampling is below 75%, it is meaningful to explore the moderating effects of other factors on the overall effect size (Stanley, Doucouliagos, Maier, & Bartoš, 2024). In this study, only 6.58% of the variance was attributed to sampling. Therefore, analyzing the potential moderating effects of study or effect size characteristics (e.g., measurement tools, gender, study quality, publication year etc.) may provide valuable insights.

A leave-one-out sensitivity analysis was performed to evaluate the robustness of the heterogeneity estimate. The I^2^ values ranged from 92.78% to 93.62%, confirming consistently high heterogeneity across the included effect sizes, with no single effect size exerting a substantial influence on the overall results. Influence diagnostics identified no outliers within the sample. The sensitivity analysis further demonstrated that r remained stable, fluctuating only between 0.25 and 0.26 when any individual sample was excluded, indicating a robust overall effect size. In terms of publication bias, the results of the 3PSM likelihood ratio test [*χ*^2^(1) = 0.28, *p* = 0.597] indicated no evidence of publication bias.

### Moderator analyses

In further analyses, we constructed ten moderation models based on study characteristics or effect sizes. We evaluated the potential moderating effects of all categorical and continuous variables, including the type of body image measure, type of risk of exercise addiction measure, type of participants, countries and regions, study design, gender, Q-SSP score, year of publication, age, and BMI. The results of the statistical analysis revealed that the type of body image measure [*F* (3, 61) = 3.13, *p* = 0.032], and the type of risk of exercise addiction measure [*F* (4, 60) = 5.63, *p* < 0.001] were significant moderating factors for the overall association between body image and risk of exercise addiction among the adult (see Supplementary Material H).

In the moderation model for the type of body image measure, the relationships between global and site-specific satisfaction (*r* = 0.25), and cognitive measures (*r* = 0.39) with risk of exercise addiction were all significant, and the relationship between cognitive measures and risk of exercise addiction was significantly higher (*β*_*1*_ = 0.15, 95% CI = 0.04–0.27) than that between global and site-specific satisfaction and risk of exercise addiction. In the moderation model for the type of risk of exercise addiction measure, the results of the relationships using the other four measures were significant, except for the using the commitment to exercise measure, and the related result using the compulsive exercise measures (*r* = 0.48) was significantly higher (*β*_*1*_ = 0.37, 95% CI = 0.21–0.53) than those using the obligatory exercise measure (*r* = 0.16).

We extended the meta-analytic model by simultaneously adding the two significant moderators mentioned above with global and site-specific satisfaction (type of body image measure) and obligatory exercise measure (type of risk of exercise addiction measure) as reference categories. The results of the statistical analysis (see Table 3) showed that the compulsive exercise measures in the type of risk of exercise addiction measure (relative to the obligatory exercise measure) had unique moderating effects (*β*_*1*_ = 0.31, 95% CI = 0.14–0.49) on the relationship between body image and risk of exercise addiction in adults.

**Table 3.** Result of multivariable moderator analyses for the association between body image and risk of exercise addiction

Moderators	*s*	*k*	*β*_*0*_	95% CI	Mean *r*	*β*_*1*_	95% CI	*F*_(*df1, df2*)_	*p*	%Var. Level 2	%Var. Level 3
	38	65						*F*_(7, 57)_ = 3.83	0.002	34.41	56.04
			0.17	0.07, 0.28	0.18						
Affective measures						−0.06	−0.22, 0.10				
Behavioral measures						0.01	−0.30, 0.32				
Cognitive measures						0.11	−0.003, 0.23				
Exercise dependence measures						0.06	−0.10, 0.21				
Compulsive exercise measures						0.31	0.14, 0.49				
Exercise addiction measures						0.10	−0.06, 0.27				
Commitment to exercise measure						−0.07	−0.35, 0.22				

*Note:* Global and site-specific satisfaction (type of body image measure) and obligatory exercise measure (type of risk of exercise addiction measure) were considered as the reference categories; Statistically significant effects (*p* < 0.05) are highlighted in bold; s = Number of studies; k = Number of effect sizes; β_0_ = Intercept/mean effect size (z); CI = Confidence interval; Mean r = mean effect size r; β_1_ = Estimated regression coefficient; F _(df1, df2)_ = Omnibus test; p = the significance test of multiple moderating effect test; %Var = percentage of variance explained.

## Discussion

Body image is considered an important variable associated with physical activity and exercise behaviors (Sabiston et al., 2019). However, the overall relationship between body image and risk of exercise addiction has not been systematically reviewed using meta-analytic techniques. The current study examined the existing quantitative evidence on the body image of multidimensional construct and risk of exercise addiction relationship in adults using a three-level random effects model, resulting in two main findings. Firstly, overall, there was a moderate positive correlation between body image and risk of exercise addiction. Secondly, the type of body image measure and the type of risk of exercise addiction measure explained the variability of this relationship. Therefore, different elements of body image and different measurement tools for risk of exercise addiction may lead to different directions and strengths in this relationship.

The findings of this study on the relationship between the body image of multidimensional construct and risk of exercise addiction further validate the applicability of the theoretical model of exercise addiction. The results reinforce the notion that exercise may become addictive through positive or negative reinforcement for body image of adults (Egorov & Szabo, 2013; Sabiston et al., 2019). The addictive exercise patterns may persist due to the positive affective experiences stemming from the belief that exercise enhances their bodies. Alternatively, such behaviors might continue as a means to avoid negative affective experiences caused by not meeting socially prescribed body ideals (Alcaraz-Ibáñez et al., 2021; Alcaraz-Ibáñez et al., 2019).

In this study, the type of body image measure was confirmed as an important moderator of in the relationship between body image and risk of exercise addiction among adults. Among the four types of body image measures, global and site-specific satisfaction and cognitive measures showed significant positive correlations with risk of exercise addiction, while affective measures and behavioral measures were not significantly associated with it. The effect size of the relationship between global and site-specific satisfaction (dissatisfaction with appearance and weight) and risk of exercise addiction aligned with findings from previous meta-analysis (Alcaraz-Ibáñez et al., 2021). Affective measures capture feelings and emotions related to body image, reflecting individuals' emotional self-awareness regarding their physical appearance and bodily function. Existing evidence indicates that participation in physical activities is associated with fewer negative and more positive body image experiences (Sabiston et al., 2019). Negative body image, including emotions such as anxiety, shame, and guilt, is often regarded as a barrier to engaging in physical exercise (Gillison, Osborn, Standage, & Skevington, 2009; Huellemann, Pila, Gilchrist, Nesbitt, & Sabiston, 2021). The affective measures used in the studies included in this review—such as the Fear of Negative Appearance Evaluation Scale, the Body Shame Subscale, and the Social Physique Anxiety Scale—primarily assess negative body image. Thus, the findings of this study are consistent with previous research in the field. Although this study suggests an insignificant relationship between behavioral measures and risk of exercise addiction, this conclusion is based on a single effect size from one study, warranting cautious interpretation. Research on body image measurement also shows that behavioral measurement tools have been underutilized (Thompson et al., 2012).

It is worth noting that cognitive measures were found to have the strongest correlation with risk of exercise addiction and have a unique moderating effect on the relationship between body image and risk of exercise addiction. Adults' thoughts and beliefs about their appearance may turn into motivations for exercise, which has been shown to predict their exercise addiction (Pritchard & Beaver, 2012). For example, people may engage in more extreme or even exercise behaviors of addictive to achieve thinness (Bonfanti et al., 2022), muscularity (Liu et al., 2018), or a fit body (Donovan et al., 2020). It has been noted that although dissatisfaction with body appearance and weight is associated with high-frequency exercise behavior, exercise motivation has an important impact on this relationship (LePage & Crowther, 2010). Cognitive measures precisely reveal people's thoughts and beliefs about their appearance, which supports the higher correlation between cognitive measures and risk of exercise addiction compared to the relationship between global and site-specific satisfaction and risk of exercise addiction in this study.

The type of risk of exercise addiction measure was also identified as a crucial moderator in the relationship between body image and risk of exercise addiction among adults. Excluding commitment to exercise measure, all four types of risk of exercise addiction measures were significantly and positively correlated with body image, with compulsive exercise measures exerting a distinct moderating effect. Systematic reviews of the prevalence of exercise addiction have proven that different assessment scales can yield different results (Marques et al., 2019; Szabo et al., 2015; Trott et al., 2020). This means that variations in risk of exercise addiction assessment tools may lead to differences in the strength and direction of the relationship between body image and risk of exercise addiction in adults. Contrarily, a meta-analysis concerning body dissatisfaction and morbid exercise behaviors presented opposing findings to this study, suggesting that the type of assessment tool does not moderate the relationship, with all four assessment tools (CET, CES, EDS-R, OEQ) showing a significant positive correlation between body dissatisfaction and morbid exercise behaviors (Alcaraz-Ibáñez et al., 2021). Given that only two effect sizes in this study utilized the commitment to exercise measure for assessing risk of exercise addiction, the relationship between body image and risk of exercise addiction assessed by this measure should be viewed with caution. An in-depth review of the Compulsive Exercise Test (CET) and Obligatory Exercise Questionnaire (OEQ) indicated that the CET includes items related to mood improvement (e.g., exercise improves my mood; I feel less anxious after I exercise), which may be related to negative emotions and ideal beliefs in body image surveys (Duncan et al., 2012; Taranis, Touyz, & Meyer, 2011). Hence, the type of risk of exercise addiction measure markedly moderates the relationship between body image and risk of exercise addiction in adults, and employing the compulsive exercise measures may yield a stronger correlation.

Furthermore, the study's examination of participant type, study region, study design, gender, age, BMI, publication time, and quality assessment score did not reveal any moderating effects on the relationship between body image and risk of exercise addiction in adults. This fact suggests that this relationship may be largely consistent among individuals with different sociodemographic characteristics. Meta-analysis on the association between body dissatisfaction and morbid exercise behavior, as well as social physique anxiety and eating disorders, corroborate these findings (Alcaraz-Ibáñez et al., 2021, 2023). The gender analysis showed that, regardless of whether participants were male or female, body image is overall related to risk of exercise addiction in adults. However, a full-text review of the included studies found that, in terms of specific body image elements, several studies (Brewster et al., 2017; Chittester & Hausenblas, 2009; Liu et al., 2018) reported a significant relationship between drive for muscularity and risk of exercise addiction in males, while related studies on females are scarce. Therefore, the evidence for the moderating role of gender in this study is limited to explaining the stable presence of the relationship between body image and risk of exercise addiction among adults of different genders. However, which specific body image elements underpin this stable relationship warrants further exploration.

It should be noted that cross-cultural studies between Poland and Indonesia revealed differentiated relationships between negative body image and risk of exercise addiction in different countries (Novita et al., 2022). Given the limited inclusion of non-Western cultural studies (*k* = 3) and significant racial differences in body image demonstrated by existing research (Gramaglia, Delicato, & Zeppegno, 2018), caution should be exercised regarding the moderating effect of the study region. A meta-analysis of the relationship between body dissatisfaction and morbid exercise behavior (based on studies from 1994 to 2019) (Alcaraz-Ibáñez et al., 2021) indicated that this relationship strengthened over publication time. However, in the present study, the moderating role of publication year was not significant, suggesting that the influence of body image on the development of risk of exercise addiction has not followed a consistent linear trend over time. Given that only one study was published in 1988, with all others published post-1998, we reanalyzed the data excluding the 1988 study. This reanalysis confirmed that the moderating role of publication year remained non-significant (*p* = 0.248). Therefore, the evidence for the moderating effect of publication year in this study is limited to explaining the stable presence of the relationship between body image and risk of exercise addiction in adults overall. However, the moderating role of publication year in the relationship between specific dimensions of body image and risk of exercise addiction requires further exploration in future research.

It is important to note that the data used for inclusion in this study are results from questionnaires that reflect trends and associations rather than diagnoses, and do not have definitive diagnostic value. Therefore, the results of this study are more appropriate for identifying potential areas of concern that require further investigation to inform the development of prevention and intervention programs targeting adolescent exercise addiction, rather than having any diagnostic value.

### Limitations and future directions

Several limitations of this study should be noted. First, most of the data comes from cross-sectional designs, and we cannot make arbitrary inferences about the bidirectional nature of the relationships between body image and risk of exercise addiction. This gap underscores the need for future longitudinal research to elucidate these complex interactions. Second, the moderation analysis is constrained by limited data for certain variables, necessitating cautious interpretation of the results. For example, only 2 studies were from non-Western cultures, only 2 studies used cross-sectional designs, and the 35 studies included covered the period from 1988 to 2022. Third, given the study design and the number of included studies, this research only reports that gender does not moderate the overall relationship between body image and risk of exercise addiction in adults. However, this does not mean that there are no gender differences in the relationships between specific body image elements and risk of exercise addiction, and further research is needed to analyze these relationships in depth. Lastly, this review is limited by the availability and quality of existing studies, which may have affected the comprehensiveness of the conclusions.

Despite these limitations, the current study has important theoretical and practical implications. First, this study confirms a positive correlation between body image and risk of exercise addiction overall in adults and that the type of body image measure is an important moderating factor. Future research should focus on more specific body image elements (e.g., specific body image behaviors, beliefs, and emotions) to analyze their relationships with risk of exercise addiction and explore gender differences. At the same time, prospective longitudinal studies should be conducted to better understand the bidirectional nature of the relationships considered. Second, different measures of risk of exercise addiction may result in different strengths of correlations. Future research should explore the definition and measurement of exercise addiction more to avoid biases resulting from inconsistent understanding and measurement. Third, although the moderating effects of the study region have been discussed, the results should be treated with caution. Future studies focusing on different regions of Asia, Latin America, and Africa would be beneficial. Fourth, given the unique moderating effect of the cognitive component of body image, developing relevant prevention and intervention programs is valuable. This may include using cognitive diagnostic techniques and cognitive-behavioral therapy for intervention, such as encouraging individuals to ignore body comparisons with others, helping individuals to develop appropriate body image beliefs, guiding individuals to receive body-related information selectively, and educating individuals to recognize negative emotions and negative body images consciously. Policymakers could use these insights to promote public health campaigns that emphasize healthy body image, reduce social pressure related to appearance, and encourage the responsible use of fitness spaces. Additionally, incorporating cognitive-behavioral strategies into community programs may help individuals build healthier exercise habits and body image beliefs.

## Conclusions

The results of this study suggest that the relationship between body image and risk of exercise addiction has a moderate positive correlation in adults and that this relationship is stronger when considering the cognitive component of body image or when risk of exercise addiction scores involve mood improvement. These findings offer valuable insights into the relationship between body image and risk of exercise addiction, encouraging exercise and health practitioners to incorporate these factors into strategies for preventing and addressing the risk of exercise addiction.

## Supplementary material

## Data Availability

Data will be made available on request.
