# Supplementary material for: Body image and risk of exercise addiction in adults: A systematic review and meta-analysis
Source: J Behav Addict. 2025 Feb 6;14(1):39–54. doi: 10.1556/2006.2024.00085 (PMC11974424; doi:10.1556/2006.2024.00085)
Supplement: Supplementary file 1 [file jba-14-039-s001.pdf]

Guo, S. et al.: Body image and risk of exercise addiction in adults: A systematic review and meta-analysis

Supplementary materials

https://doi.org/10.1556/2006.2024.00085

Supplementary Material A PRISMA 2020 Checklist

Section and Topic	Item #	Checklist item	Location where item is reported
**TITLE**			
Title	1	Identify the report as a systematic review.	1
**ABSTRACT**			
Abstract	2	See the PRISMA 2020 for Abstracts checklist.	1*
**INTRODUCTION**			
Rationale	3	Describe the rationale for the review in the context of existing knowledge.	1-2
Objectives	4	Provide an explicit statement of the objective(s) or question(s) the review addresses.	2-3
**METHODS**			
Eligibility criteria	5	Specify the inclusion and exclusion criteria for the review and how studies were grouped for the syntheses.	3-4
Information sources	6	Specify all databases, registers, websites, organisations, reference lists and other sources searched or consulted to identify studies. Specify the date when each source was last searched or consulted.	3
Search strategy	7	Present the full search strategies for all databases, registers and websites, including any filters and limits used.	3, Appendix B
Selection process	8	Specify the methods used to decide whether a study met the inclusion criteria of the review, including how many reviewers screened each record and each report retrieved, whether they worked independently, and if applicable, details of automation tools used in the process.	3-4
Data collection process	9	Specify the methods used to collect data from reports, including how many reviewers collected data from each report, whether they worked independently, any processes for obtaining or confirming data from study investigators, and if applicable, details of automation tools used in the process.	4
Data items	10a	List and define all outcomes for which data were sought. Specify whether all results that were compatible with each outcome domain in each study were sought (e.g. for all measures, time points, analyses), and if not, the methods used to decide which results to collect.	5, Appendix C,
	10b	List and define all other variables for which data were sought (e.g. participant and intervention characteristics, funding sources). Describe any assumptions made about any missing or unclear information.	NA
Study risk of bias assessment	11	Specify the methods used to assess risk of bias in the included studies, including details of the tool(s) used, how many reviewers assessed each study and whether they worked independently, and if applicable, details of automation tools used in the process.	5, Appendix E
Effect measures	12	Specify for each outcome the effect measure(s) (e.g. risk ratio, mean difference) used in the synthesis or presentation of results.	5-6
Synthesis methods	13a	Describe the processes used to decide which studies were eligible for each synthesis (e.g. tabulating the study intervention characteristics and comparing against the planned groups for each synthesis (item #5)).	4, Appendix D
	13b	Describe any methods required to prepare the data for presentation or synthesis, such as handling of missing summary statistics, or data conversions.	5-6
	13c	Describe any methods used to tabulate or visually display results of individual studies and syntheses.	6
	13d	Describe any methods used to synthesize results and provide a rationale for the choice(s). If meta-analysis was performed, describe the model(s), method(s) to identify the presence and extent of statistical heterogeneity, and software package(s) used.	5-6
	13e	Describe any methods used to explore possible causes of heterogeneity among study results (e.g. subgroup analysis, meta-regression).	5-6
	13f	Describe any sensitivity analyses conducted to assess robustness of the synthesized results.	6

Supplementary Material A PRISMA 2020 Checklist

Section and Topic	Item #	Checklist item	Location where item is reported
Reporting bias assessment	14	Describe any methods used to assess risk of bias due to missing results in a synthesis (arising from reporting biases).	6
Certainty assessment	15	Describe any methods used to assess certainty (or confidence) in the body of evidence for an outcome.	6
**RESULTS**			
Study selection	16a	Describe the results of the search and selection process, from the number of records identified in the search to the number of studies included in the review, ideally using a flow diagram.	6-7, Figure 1
	16b	Cite studies that might appear to meet the inclusion criteria, but which were excluded, and explain why they were excluded.	6-7, Figure 1
Study characteristics	17	Cite each included study and present its characteristics.	Table 2, Appendix F
Risk of bias in studies	18	Present assessments of risk of bias for each included study.	9
Results of individual studies	19	For all outcomes, present, for each study: (a) summary statistics for each group (where appropriate) and (b) an effect estimate and its precision (e.g. confidence/credible interval), ideally using structured tables or plots.	8-12, Table 2-3
Results of syntheses	20a	For each synthesis, briefly summarise the characteristics and risk of bias among contributing studies.	7-8
	20b	Present results of all statistical syntheses conducted. If meta-analysis was done, present for each the summary estimate and its precision (e.g. confidence/credible interval) and measures of statistical heterogeneity. If comparing groups, describe the direction of the effect.	8-12, Table 2-3
	20c	Present results of all investigations of possible causes of heterogeneity among study results.	Appendix F
	20d	Present results of all sensitivity analyses conducted to assess the robustness of the synthesized results.	14
Reporting biases	21	Present assessments of risk of bias due to missing results (arising from reporting biases) for each synthesis assessed.	14
Certainty of evidence	22	Present assessments of certainty (or confidence) in the body of evidence for each outcome assessed.	14-15
**DISCUSSION**			
Discussion	23a	Provide a general interpretation of the results in the context of other evidence.	15
	23b	Discuss any limitations of the evidence included in the review.	16-18
	23c	Discuss any limitations of the review processes used.	18
	23d	Discuss implications of the results for practice, policy, and future research.	18-19
**OTHER INFORMATION**			
Registration and protocol	24a	Provide registration information for the review, including register name and registration number, or state that the review was not registered.	3
	24b	Indicate where the review protocol can be accessed, or state that a protocol was not prepared.	3
	24c	Describe and explain any amendments to information provided at registration or in the protocol.	3
Support	25	Describe sources of financial or non-financial support for the review, and the role of the funders or sponsors in the review.	NA
Competing interests	26	Declare any competing interests of review authors.	19

Supplementary Material A PRISMA 2020 Checklist

Section and Topic	Item #	Checklist item	Location where item is reported
Availability of data, code and other materials	27	Report which of the following are publicly available and where they can be found: template data collection forms; data extracted from included studies; data used for all analyses; analytic code; any other materials used in the review.	19

*The requirements listed in the Abstract checklist were only partially fulfilled due to space limitations.

Supplementary Material B

Search strategy

MEDLINE

1. Body Image/
2. ((awareness or affect or appreciat* or areas satisfaction or assessment or behav* or critici* or cognition* or concern* or discrepancy or dissatisfaction or distortion or disturbance or drive for muscularity or embarrass* or envy or envious or experience? or function* or guilt or perception? or pride or proud or project* or representation* or questionnaire? or schema* or shame* or satisfaction or scale?) adj2 (appearance or body)).tw,kf.
3. ((concern* or internalization* or embarrassment or emotion* or envy or envious or guilt or pride or shame) adj2 weight).tw,kf.
4. (objectification or physical appearance or physical attractiveness).tw,kf.
5. body image.tw,kf.
6. ((embarrassment or envy or shame or pride or guilt*) adj2 body-related).tw,kf.
7. 1 or 2 or 3 or 4 or 5 or 6 N=64580
8. Compulsive Exercise/
9. (obligatory exercise* or exercise dependence or exercise addiction or compulsive exercise or sport dependence or sport addiction or obligatory sport or physical activity dependence or physical activity addiction or obligatory physical activity or physical activity compulsive or run* dependence or run* addiction or obligatory run* or compulsive run* or bigorexia or excessive exercise* or unhealth exercise* or exercise abuse or exercise pathology or pathological exercise or overexercise* or over-exercise* or compulsive exercising or compulsive sporting or sports addiction)
10. 8 or 9 N=1467
11. 7 and 10 N=311

Embase

1. 'body image'/exp OR 'body image'
2. (((awareness OR affect OR appreciat* OR areas) AND satisfaction OR assessment OR behav* OR critici* OR cognition* OR concern* OR discrepancy OR dissatisfaction OR distortion OR disturbance OR drive) AND for AND muscularity OR embarrass* OR envy OR envious OR experience? OR function* OR guilt OR perception? OR pride OR proud OR project* OR representation* OR questionnaire? OR schema* OR shame* OR satisfaction OR scale?) AND (appearance OR body)
3. (concern* OR internalization* OR embarrassment OR emotion* OR envy OR envious OR guilt OR pride OR shame) AND weight
4. ((objectification OR physical) AND appearance OR physical) AND attractiveness
5. body AND image
6. (embarrassment OR envy OR shame OR pride OR guilt*) AND 'body related'
7. 1 OR 2 OR 3 OR 4 OR 5 OR 6 N=1068825

8. 'exercise addiction'/exp OR 'exercise addiction'
9. 'obligatory exercise*' OR 'exercise dependence' OR 'exercise addiction' OR 'compulsive exercise' OR 'sport dependence' OR 'sport addiction' OR 'obligatory sport' OR 'physical activity dependence' OR 'physical activity addiction' OR 'obligatory physical activity' OR 'physical activity compulsive' OR 'run* dependence' OR 'run* addiction' OR 'obligatory run*' OR 'compulsive run*' OR bigorexia OR 'excessive exercise*' OR 'unhealth exercise*' OR 'exercise abuse' OR 'exercise pathology' OR 'pathological exercise' OR overexercise* OR 'over exercise*' OR 'compulsive exercising' OR 'compulsive sporting' OR 'sports addiction'
10. 8 OR 9 N=1956
11. 7 AND 10 N=590

PsycINFO
S1. MA body image OR AB ((awareness or affect or appreciat* or areas satisfaction or assessment or behav* or critici* or cognition* or concern* or discrepancy or dissatisfaction or distortion or disturbance or drive for muscularity or embarrass* or envy or envious or experience? or function* or guilt or perception? or pride or proud or project* or representation* or questionnaire? or schema* or shame* or satisfaction or scale?) and (appearance or body)) OR AB ((concern* or internalization* or embarrassment or emotion* or envy or envious or guilt or pride or shame) and weight) OR AB (((objectification OR physical) AND appearance OR physical) AND attractiveness) OR AB body image OR AB ((embarrassment OR envy OR shame OR pride OR guilt*) AND 'body related')
S2. MA compulsive exercise OR AB (obligatory exercise* or exercise dependence or exercise addiction or compulsive exercise or sport dependence or sport addiction or obligatory sport or physical activity dependence or physical activity addiction or obligatory physical activity or physical activity compulsive or run* dependence or run* addiction or obligatory run* or compulsive run* or bigorexia or excessive exercise* or unhealth exercise* or exercise abuse or exercise pathology or pathological exercise or overexercise* or over-exercise* or compulsive exercising or compulsive sporting or sports addiction)
S3. S1 AND S2 N=572

SPORT Discus
S1. SU body image OR AB ((awareness or affect or appreciat* or areas satisfaction or assessment or behav* or critici* or cognition* or concern* or discrepancy or dissatisfaction or distortion or disturbance or drive for muscularity or embarrass* or envy or envious or experience? or function* or guilt or perception? or pride or proud or project* or representation* or questionnaire? or schema* or shame* or satisfaction or scale?) and (appearance or body)) OR AB ((concern* or internalization* or embarrassment or emotion* or envy or envious or guilt or pride or shame) and weight)

OR AB (((objectification OR physical) AND appearance OR physical) AND attractiveness) OR AB body image OR AB ((embarrassment OR envy OR shame OR pride OR guilt*) AND 'body related')
S2. SU compulsive exercise OR AB (obligatory exercise* or exercise dependence or exercise addiction or compulsive exercise or sport dependence or sport addiction or obligatory sport or physical activity dependence or physical activity addiction or obligatory physical activity or physical activity compulsive or run* dependence or run* addiction or obligatory run* or compulsive run* or bigorexia or excessive exercise* or unhealth exercise* or exercise abuse or exercise pathology or pathological exercise or overexercise* or over-exercise* or compulsive exercising or compulsive sporting or sports addiction)
S3. S1 AND S2 N=531

Web of Science
1. ((((TS=(body image)) OR TS=((awareness or affect or appreciat* or areas satisfaction or assessment or behav* or critici* or cognition* or concern* or discrepancy or dissatisfaction or distortion or disturbance or drive for muscularity or embarrass* or envy or envious or experience? or function* or guilt or perception? or pride or proud or project* or representation* or questionnaire? or schema* or shame* or satisfaction or scale?) and (appearance or body))) OR TS=((concern* or internalization* or embarrassment or emotion* or envy or envious or guilt or pride or shame) and weight)) OR TS=(((objectification OR physical) AND appearance OR physical) AND attractiveness)) OR TS=((embarrassment OR envy OR shame OR pride OR guilt*) AND 'body related')
2. (TS=(compulsive exercise)) OR TS=(obligatory exercise* or exercise dependence or exercise addiction or compulsive exercise or sport dependence or sport addiction or obligatory sport or physical activity dependence or physical activity addiction or obligatory physical activity or physical activity compulsive or run* dependence or run* addiction or obligatory run* or compulsive run* or bigorexia or excessive exercise* or unhealth exercise* or exercise abuse or exercise pathology or pathological exercise or overexercise* or over-exercise* or compulsive exercising or compulsive sporting or sports addiction)
3. 1 AND 2 N=4678

Supplementary Mterial C

Extraction Form

Paper_ID	ID of included papers
Study_ID	ID of the study in a paper
Effectsize_ID	ID of included effect sizes
Authors/Year	Name of authors / publication year
Title	Title of the paper
Year	Publication year
Source	Publication Title
Region	Region of the studied sample
Gender/N	Gender of the sample
Mean age (SD or range)	Mean age (Standard Deviation or range) of the sample
MeanBMI	Mean Body mass index of the sample
Population	Characteristics of the sample
Sampling_Category	Sampling method of samples
Body image measure	Measurement tool of body image
Risk of exercise addiction measure	Measurement tool of risk of exercise addiction
Study design	Cross-sectional or Longitudinal
Correlation_r	Pearson correlation coefficient

Supplementary Material D

The types of body image measures and risk of exercise addiction measures for included studies

Variable	Name of type	Measure	Included studies
Body image	Global and site-specific satisfaction	Body Cathexis Scale (Tucker, 1981)	P8S1
		Body Image and Body Change Inventory - Body Image Concern subscale (Ricciardelli & McCabe, 2002)	P17S1; P22S1
		Body Esteem Scale (Mendelson et al., 2001)	P38S1
		Body Image Concern inventory - dysmorphic symptoms subscale (Littleton et al., 2005)	P29S2; P30S1; P36S1; P37S1
		Body Parts Satisfaction Scale for Men (McFarland & Petrie, 2012)	P18S2
		Body Self Relations Questionnaire - Physical Appearance Evaluation Subscale (Noles et al., 1985)	P1S2; P1S4; P1S6; P1S8
		Body Shape Questionnaire (Cooper et al., 1987; Evans & Dolan, 1993)	P15S1; P15S2; P20S1; P27S1
		Body-Image Ideals Questionnaire (Cash & Szymanski, 1995)	P14S3; P24S2; P32S3
		Body Uneasiness Test - Body Image Concerns Subscale (Cuzzolaro et al., 2006)	P35S1
		Eating Disorder Examination Questionnaire – Shape and Weight Concern Subscales (Fairburn & Beglin, 1994)	P10S1; P10S2; P34S1; P34S2
		Eating Disorder Inventory / 2 / 3 - Body Dissatisfaction Subscale (Garner, 2004; Garner, 1991; Garner et al., 1983)	P1S1; P1S3; P1S5; P1S7; P3S1; P4S1; P5S1; P13S1; P29S1

		Eating Pathology Symptoms Inventory - Body Dissatisfaction subscale (Forbush et al., 2013)	P19S1
		Male Body Attitudes Scale - Body Fat and Muscle Dissatisfaction (Tylka et al., 2005)	P26S1; P26S2
		Multidimensional Body Self-Relations Questionnaire - Appearance Evaluation and Body Areas Satisfaction Scales (Brown et al., 1990)	P11S1; P11S2; P12S1; P28S1
		Muscular Figure Rating Scale (Peters & Phelps, 2001)	P23S1
		Revised Male Body Image Attitudes Scale - Body Fat and Muscularity Subscale (Ryan et al., 2011)	P25S1; P25S2
	Affective measure	Fear of Negative Appearance Evaluation Scale (Lundgren et al., 2004)	P33S1; P33S2
		Objectified Body Consciousness Scale - Body Shame Subscale (McKinley & Hyde, 1996)	P21S1
		Social Physique Anxiety Scale (Hart et al., 1989)	P2S1; P6S1; P6S2; P7S1; P7S2; P16S1
	Behavioral measures	Body Image Concern Inventory - symptom interference subscale (Littleton et al., 2005)	P30S2
	Cognitive measures	Appearance Schemas Inventory Revised - Motivational and self-evaluative Salience Subscale (Cash et al., 2004)	P14S1; P14S2
		Drive for Leanness Scale (Smolak & Murnen, 2008)	P31S1
		Drive for Muscularity Scale (McCreary & Sasse, 2000)	P9S1; P9S2; P18S3; P23S2
		Fit Ideal Internalization Test (Uhlmann et al., 2020)	P24S1; P32S1

		Objectified Body Consciousness Scale - Body Surveillance subscale (McKinley & Hyde, 1996)	P18S1; P32S2
Risk of exercise addiction	Obligatory exercise measure	Obligatory Exercise Questionnaire (OEQ) (Pasman & Thompson, 1988)	P1S1; P1S2; P1S3; P1S4; P1S5; P1S6; P1S7; P1S8; P2S1; P3S1; P4S1; P5S1; P6S1; P6S2; P7S1; P7S2; P10S1; P10S2; P11S1; P11S2; P12S1; P13S1; P15S1; P15S2; P17S1; P22S1; P33S1; P33S2
	Exercise dependence measures	Exercise Dependence Scale / Revised (EDS / EDS-R) (Downs et al., 2004; Hausenblas & Downs, 2002)	P9S1; P9S2; P14S1; P14S2; P14S3; P16S1; P23S1; P23S2; P25S1; P25S2; P26S1; P26S2; P28S1; P34S1; P34S2
	Compulsive exercise measures	Compulsive Exercise Test (CET) (Taranis et al., 2011); Exercise and Eating Disorder Questionnaire (EED) - Compulsive and Positive and Healthy Exercise Subscales (Danielsen et al., 2015)	P18S1; P18S2; P18S3; P20S1; P24S1; P24S2; P27S1; P32S1; P32S2; P32S3; P35S1
	Exercise addiction measures	Exercise Addiction Inventory (EAI) (Griffiths et al., 2005; Terry et al., 2004)	P21S1; P29S1; P29S2; P30S1; P30S2; P31S1; P36S1; P37S1; P38S1
	Commitment to exercise measure	Commitment to Exercise Scale (CES) (Davis et al., 1993)	P8S1; P19S1

Supplementary Material E

The Quality of Survey Studies in Psychology (Q-SSP) checklist (Protogerou & Hagger, 2020)

Identity	Types	Items
I1	Introduction	Was the problem or phenomenon under investigation defined, described, and justified?
I2		Was the population under investigation defined, described, and justified?
I3		Were specific research questions or hypotheses stated?
I4		Were operational definitions of all study variables provided?
P1	Participants	Were participant inclusion criteria stated?
P2		Was the participant recruitment strategy described?
P3		Was a justification/rationale for the sample size provided?
D1	Data	Was the attrition rate provided? (applies to cross-sectional and prospective studies)
D2		Was a method of treating attrition provided? (applies to cross-sectional and prospective studies)
D3		Were the data analysis techniques justified (i.e., was the link between hypotheses/aims/research questions and data analyses explained)?
D4		Were the measures provided in the report (or in a supplement) in full?
D5		Was evidence provided for the validity of all the measures (or instrument) used?
D6		Was information provided about the person(s) who collected the data (e.g., training, expertise, other demographic characteristics)?
D7		Was information provided about the context (e.g., place) of data collection?
D8		Was information provided about the duration (or start and end date) of data collection?
D9		Was the study sample described in terms of key demographic characteristics?
D10		Was discussion of findings confined to the population from which the sample was drawn?
E1	Ethics	Were participants asked to provide (informed) consent or assent?
E2		Were participants debriefed at the end of data collection?
E3		Were funding sources or conflicts of interest disclosed?

Q-SSP scores of the studies included in the present study

Paper ID	I1	I2	I3	I4	P1	P2	P3	D1	D2	D3	D4	D5	D6	D7	D8	D9	D10	E1	E2	E3	Total YES	Total Non NA	Percentage YES	Rating
P1	Yes	Yes	Yes	Yes	Yes	Yes	No	Yes	Yes	Yes	Yes	Yes	No	Yes	No	Yes	Yes	Yes	NA	No	15	19	78.95	Acceptable quality
P2	Yes	Yes	Yes	Yes	Yes	Yes	No	No	Yes	Yes	Yes	Yes	No	Yes	No	Yes	Yes	Yes	NA	No	14	19	73.68	Acceptable quality
P3	Yes	Yes	Yes	Yes	Yes	Yes	No	No	No	Yes	Yes	Yes	No	Yes	Yes	Yes	Yes	Yes	NA	No	14	19	73.68	Acceptable quality
P4	Yes	Yes	Yes	Yes	Yes	Yes	No	Yes	Yes	Yes	Yes	Yes	No	Yes	No	Yes	Yes	Unclear	NA	No	14	19	73.68	Acceptable quality
P5	Yes	Yes	Yes	Yes	Yes	Yes	No	No	Yes	Yes	Yes	Yes	No	Yes	No	Yes	Yes	Unclear	NA	No	13	19	68.42	Questionable quality
P6	Yes	Yes	Yes	Yes	Yes	Yes	No	Yes	Yes	Yes	Yes	Yes	No	Yes	No	Yes	Yes	Yes	NA	Yes	16	19	84.21	Acceptable quality
P7	Yes	Yes	Yes	Yes	Yes	Yes	No	Yes	Yes	Yes	Yes	Yes	No	Yes	Yes	Yes	Yes	Yes	NA	Yes	17	19	89.47	Acceptable quality
P8	Yes	Yes	Yes	Yes	Yes	Yes	No	Yes	Yes	Yes	Yes	Yes	No	Yes	Yes	Yes	Yes	Yes	NA	No	16	19	84.21	Acceptable quality
P9	Yes	Yes	Yes	Yes	Yes	Yes	Yes	Yes	Yes	Yes	Yes	Yes	No	Yes	Yes	Yes	Yes	Yes	NA	Yes	18	19	94.74	Acceptable quality
P10	Yes	Yes	Yes	Yes	Yes	Yes	No	No	No	Yes	Yes	Yes	No	Yes	Yes	Yes	Yes	Unclear	NA	Yes	14	19	73.68	Acceptable quality
P11	Yes	Yes	Yes	Yes	Yes	Yes	No	No	No	Yes	Yes	Yes	NA	NA	Yes	Yes	Yes	Yes	NA	No	13	17	76.47	Acceptable quality
P12	Yes	Yes	Yes	Yes	Yes	Yes	No	Yes	Yes	Yes	Yes	Yes	No	Yes	Yes	Yes	Yes	Yes	NA	Yes	17	19	89.47	Acceptable

																								quality
P13	Yes	Yes	Yes	Yes	Yes	Yes	No	Yes	Yes	Yes	Yes	Yes	NA	NA	Yes	Yes	Yes	Unclear	NA	Yes	15	17	88.24	Acceptable quality
P14	Yes	Yes	Yes	Yes	Yes	Yes	No	Yes	Yes	Yes	Yes	Yes	No	Yes	Yes	Yes	Yes	Yes	NA	No	16	19	84.21	Acceptable quality
P15	Yes	Yes	Yes	Yes	Yes	Yes	No	Yes	Yes	Yes	Yes	Yes	No	Yes	Yes	Yes	Yes	Yes	NA	No	16	19	84.21	Acceptable quality
P16	Yes	Yes	Yes	Yes	Yes	Yes	No	Yes	Yes	Yes	Yes	Yes	NA	NA	Yes	Yes	Yes	Yes	NA	Yes	16	17	94.12	Acceptable quality
P17	Yes	Yes	Yes	Yes	Yes	Yes	No	No	No	Yes	Yes	Yes	NA	NA	No	Yes	Yes	Yes	NA	Yes	13	17	76.47	Acceptable quality
P18	Yes	Yes	Yes	Yes	Yes	Yes	No	Yes	Yes	Yes	Yes	Yes	NA	NA	No	Yes	Yes	Yes	NA	No	14	17	82.35	Acceptable quality
P19	Yes	Yes	Yes	Yes	Yes	Yes	No	No	No	Yes	Yes	Yes	NA	NA	Yes	Yes	Yes	Yes	NA	No	13	17	76.47	Acceptable quality
P20	Yes	Yes	Yes	Yes	Yes	Yes	No	Yes	Yes	Yes	Yes	Yes	No	Yes	No	Yes	Yes	Yes	NA	Yes	16	19	84.21	Acceptable quality
P21	Yes	Yes	Yes	Yes	Yes	Yes	No	No	No	Yes	Yes	Yes	NA	NA	No	Yes	Yes	Yes	NA	Yes	13	17	76.47	Acceptable quality
P22	Yes	Yes	Yes	Yes	No	Yes	No	No	No	Yes	Yes	Yes	NA	NA	No	Yes	Yes	Yes	NA	Yes	12	17	70.59	Acceptable quality
P23	Yes	Yes	Yes	Yes	Yes	Yes	No	Yes	Yes	Yes	Yes	Yes	No	Yes	Yes	Yes	Yes	Yes	NA	Yes	17	19	89.47	Acceptable quality
P24	Yes	Yes	Yes	Yes	No	Yes	No	No	No	Yes	Yes	Yes	NA	NA	No	Yes	Yes	Yes	NA	Yes	12	17	70.59	Acceptable quality
P25	Yes	Yes	Yes	Yes	Yes	Yes	No	Yes	Yes	Yes	Yes	Yes	NA	NA	Yes	Yes	Yes	Yes	NA	Yes	16	17	94.12	Acceptable quality

P26	Yes	Yes	Yes	Yes	No	Yes	No	Yes	Yes	Yes	Yes	Yes	NA	NA	No	Yes	Yes	Yes	NA	Yes	14	17	82.35	Acceptable quality
P27	Yes	Yes	Yes	Yes	Yes	Yes	No	Yes	Yes	Yes	Yes	Yes	No	Yes	No	Yes	Yes	Yes	NA	Yes	16	19	84.21	Acceptable quality
P28	Yes	Yes	Yes	Yes	No	Yes	No	No	No	Yes	Yes	Yes	Yes	Yes	Yes	Yes	Yes	Yes	NA	Yes	15	19	78.95	Acceptable quality
P29	Yes	Yes	Yes	Yes	Yes	Yes	No	No	No	Yes	Yes	Yes	NA	NA	No	Yes	Yes	Yes	NA	Yes	13	17	76.47	Acceptable quality
P30	Yes	Yes	Yes	Yes	Unclear	Yes	No	No	No	Yes	Yes	Yes	NA	NA	No	Yes	Yes	Yes	NA	Yes	12	17	70.59	Acceptable quality
P31	Yes	Yes	Yes	Yes	Unclear	Yes	No	Yes	Yes	Yes	No	Yes	NA	NA	Yes	Yes	Yes	Yes	NA	Yes	14	17	82.35	Acceptable quality
P32	Yes	Yes	Yes	Yes	No	Yes	No	No	No	Yes	Yes	Yes	NA	NA	No	Yes	Yes	Yes	NA	Yes	12	17	70.59	Acceptable quality
P33	Yes	Yes	Yes	Yes	No	Yes	No	No	No	Yes	Yes	Yes	NA	NA	Yes	Yes	Yes	Yes	NA	Yes	13	17	76.47	Acceptable quality
P34	Yes	Yes	Yes	Yes	Yes	Yes	No	No	No	Yes	Yes	Yes	NA	NA	No	Yes	Yes	Unclear	NA	Yes	12	17	70.59	Acceptable quality
P35	Yes	Yes	Yes	Yes	Yes	Yes	No	Yes	Yes	Yes	Yes	Yes	NA	NA	No	Yes	Yes	Yes	NA	Yes	15	17	88.24	Acceptable quality
P36	Yes	Yes	Yes	Yes	Yes	Yes	No	Yes	Yes	Yes	Yes	Yes	NA	NA	No	Yes	Yes	Yes	NA	Yes	15	17	88.24	Acceptable quality
P37	Yes	Yes	Yes	Yes	Yes	Yes	No	No	No	Yes	Yes	Yes	NA	NA	No	Yes	Yes	Yes	NA	Yes	13	17	76.47	Acceptable quality
P38	Yes	Yes	Yes	Yes	Yes	Yes	Yes	Yes	Yes	Yes	Yes	Yes	NA	NA	Yes	Yes	Yes	Yes	NA	Yes	17	17	100	Acceptable quality

Supplementary Material F

Characteristics of the studies included in the present study

Study_ID	Authors_Year	Title	Region	Gender/N	Mean age (SD or range)	MeanBMI	Population	Sampling_Category	Body image measure	Risk of exercise addiction measure	Study design	Correlation_r
P1S1	Pasman & Thompson_1988	Body image and eating disturbance in obligatory runners, obligatory weightlifters, and sedentary individuals	USA	F/30	NA	NA	Runners	meet	Eating Disorder Inventory (EDI) – Body Dissatisfaction Subscale	Obligatory Exercise Questionnaire (OEQ)	Cross-sectional	0.125
P1S2	Pasman & Thompson_1988	Body image and eating disturbance in obligatory runners, obligatory weightlifters, and sedentary individuals	USA	F/30	NA	NA	Runners	meet	Body Self Relations Questionnaire - Physical Appearance Evaluation Subscale	Obligatory Exercise Questionnaire (OEQ)	Cross-sectional	0.21
P1S3	Pasman & Thompson_1988	Body image and eating disturbance in obligatory runners, obligatory weightlifters, and sedentary individuals	USA	F/30	NA	NA	exercisers in fitness centers	meet	Eating Disorder Inventory (EDI) – Body Dissatisfaction Subscale	Obligatory Exercise Questionnaire (OEQ)	Cross-sectional	-0.185
P1S4	Pasman & Thompson_1988	Body image and eating disturbance in obligatory runners, obligatory weightlifters, and sedentary individuals	USA	F/30	NA	NA	exercisers in fitness centers	meet	Body Self Relations Questionnaire - Physical Appearance Evaluation Subscale	Obligatory Exercise Questionnaire (OEQ)	Cross-sectional	-0.174
P1S5	Pasman &	Body image and eating disturbance in obligatory runners, obligatory weightlifters, and sedentary	USA	M/30	NA	NA	Runners	meet	Eating Disorder Inventory (EDI) – Body Dissatisfaction	Obligatory Exercise	Cross-sectional	0.238

	Thompson_1988	individuals							Subscale	Questionnaire (OEQ)		
P1S6	Pasman & Thompson_1988	Body image and eating disturbance in obligatory runners, obligatory weightlifters, and sedentary individuals	USA	M/30	NA	NA	Runners	meet	Body Self Relations Questionnaire - Physical Appearance Evaluation Subscale	Obligatory Exercise Questionnaire (OEQ)	Cross-sectional	-0.082
P1S7	Pasman & Thompson_1988	Body image and eating disturbance in obligatory runners, obligatory weightlifters, and sedentary individuals	USA	M/30	NA	NA	exercisers in fitness centers	meet	Eating Disorder Inventory (EDI) – Body Dissatisfaction Subscale	Obligatory Exercise Questionnaire (OEQ)	Cross-sectional	-0.117
P1S8	Pasman & Thompson_1988	Body image and eating disturbance in obligatory runners, obligatory weightlifters, and sedentary individuals	USA	M/30	NA	NA	exercisers in fitness centers	meet	Body Self Relations Questionnaire - Physical Appearance Evaluation Subscale	Obligatory Exercise Questionnaire (OEQ)	Cross-sectional	-0.263
P2S1	Diehl et al._1998	Social physique anxiety and disordered eating: what's the connection?	USA	F/160	21.53 (3.95)	22.22	General university students	undergraduate psychology classes	Social Physique Anxiety Scale (SPAS)	Obligatory Exercise Questionnaire (OEQ)	Cross-sectional	0.06
P3S1	Gulker et al._2001	Do excessive exercisers have a higher rate of obsessive-compulsive symptomatology?	USA	F&M/192	36 (18-81)	NA	Regular exercisers	meet a researcher	Eating Disorder Inventory 2 (EDI 2) - Body Dissatisfaction Subscale	Obligatory Exercise Questionnaire (OEQ)	Cross-sectional	0.201
P4S1	Mussap_2006	Reinforcement sensitivity theory (RST) and body change behaviour in males	Australia	M/120	25.94 (18-40)	24.35	General university students	NA	Eating Disorder Inventory 2(EDI 2) – Body Dissatisfaction Subscale	Obligatory Exercise Questionnaire	Cross-sectional	0.29

										(OEQ)		
P5S1	Mussap_ 2007	Motivational processes associated with unhealthy body change attitudes and behaviours	Australia	F/130	25.1 (18-40)	NA	General university students	NA	Eating Disorder Inventory 2(EDI 2) – Body Dissatisfaction Subscale	Obligatory Exercise Questionnaire (OEQ)	Cross-sectional	0.37
P6S1	Chu et al._2008	Social physique anxiety, obligation to exercise, and exercise choices among college students	USA	F/200	NA	NA	General university students	meet	Social Physique Anxiety Scale (SPAS)	Obligatory Exercise Questionnaire (OEQ)	Cross-sectional	0.09
P6S2	Chu et al._2008	Social physique anxiety, obligation to exercise, and exercise choices among college students	USA	M/137	NA	NA	General university students	meet	Social Physique Anxiety Scale (SPAS)	Obligatory Exercise Questionnaire (OEQ)	Cross-sectional	0.06
P7S1	Bushman et al._2009	Social Physique Anxiety and Obligation to Exercise in College Males and Females: Exercise Activity, Location, and Partners	USA	F/170	NA	NA	General university students	conducted at the onset of class	Social Physique Anxiety Scale (SPAS)	Obligatory Exercise Questionnaire (OEQ)	Cross-sectional	0.017
P7S2	Bushman et al._2009	Social Physique Anxiety and Obligation to Exercise in College Males and Females: Exercise Activity, Location, and Partners	USA	M/141	NA	NA	General university students	conducted at the onset of class	Social Physique Anxiety Scale (SPAS)	Obligatory Exercise Questionnaire (OEQ)	Cross-sectional	0.06
P8S1	Fortier et al._2009	Comparing self-determination and body image between excessive and healthy exercisers	Canada	F&M/110	NA	NA	Regular exercisers	completed at club facilities after	Body Cathexis Scale	Commitment to Exercise Scale (CES); 12-Month Leisure-Time	Cross-sectional	0.127

								workouts /training		Physical Activity History		
P9S1	Chittester al._2009	Correlates of drive for muscularity: The role of anthropometric measures and psychological factors	USA	M/113	20.34 (1.52)	25.05	General university students	met at the Exercise Psychology Lab	Drive for Muscularity Scale - Muscle-oriented body image subscale	Exercise Dependence Scale (EDS)	Cross-sectional	0.35
P9S2	Chittester al._2009	Correlates of drive for muscularity: The role of anthropometric measures and psychological factors	USA	M/113	20.34 (1.52)	25.05	General university students	met at the Exercise Psychology Lab	Drive for Muscularity Scale - Muscularity-related behavior subscale	Exercise Dependence Scale (EDS)	Cross-sectional	0.57
P10S1	De Young & Anderson _2010	The importance of the function of exercise in the relationship between obligatory exercise and eating and body image concerns	USA	F&M/226	19.3 (2.6)	NA	General university students	a course requirement.	Eating Disorder Examination Questionnaire (EDE-Q) – Shape Concern Subscales	Obligatory Exercise Questionnaire (OEQ)	Cross-sectional	0.275
P10S2	De Young & Anderson _2010	The importance of the function of exercise in the relationship between obligatory exercise and eating and body image concerns	USA	F&M/226	19.3 (2.6)	NA	General university students	a course requirement.	Eating Disorder Examination Questionnaire (EDE-Q) – Weight Concern Subscales	Obligatory Exercise Questionnaire (OEQ)	Cross-sectional	0.236
P11S1	Boroughs et al._2010	Body dysmorphic disorder among diverse racial/ethnic and sexual orientation groups: Prevalence estimates and associated factors	USA	F&M/1041	20.95 (18-56)	NA	General university students	online	Multidimensional Body Self-Relations Questionnaire (MBSRQ) - Appearance Evaluation Scale	Obligatory Exercise Questionnaire (OEQ)	Cross-sectional	0.01
P11S2	Boroughs	Body dysmorphic disorder among diverse	USA	F&M/1	20.95	NA	General	online	Multidimensional Body	Obligatory	Cross-sec	-0.02

	et al._2010	racial/ethnic and sexual orientation groups: Prevalence estimates and associated factors		041	(18-56)		university students		Self-Relations Questionnaire (MBSRQ) - Body Areas Satisfaction Scale	Exercise Questionnaire (OEQ)	tional	
P12S1	Homan_2010	Athletic-ideal and thin-ideal internalization as prospective predictors of body dissatisfaction, dieting, and compulsive exercise	USA	F/231	19.2 (1.1)	22	General university students	completed the measures in a classroom setting	Multidimensional Body-Self Relations Questionnaire (MBSRQ) – Body Areas Satisfaction Subscales	Obligatory Exercise Questionnaire (OEQ)	Longitudinal	0.36
P13S1	Gapin & Petruzzello_2011	Athletic identity and disordered eating in obligatory and non-obligatory runners	USA	F&M/179	35.88 (18-67)	22.71	Runners	meet or email	Eating Disorder Inventory (EDI) – Body Dissatisfaction Scale	Obligatory Exercise Questionnaire (OEQ)	Cross-sectional	0.22
P14S1	Lamarche & Gammage_2012	Predicting exercise and eating behaviors from appearance evaluation and two types of investment	Canada	F/231	20.92 (2.62)	22.98	General university students	at the laboratory	Appearance Schemas Inventory-Revised (ASI-R) - self-evaluative salience Subscale	Exercise Dependence Scale (EDS)	Cross-sectional	0.26
P14S2	Lamarche & Gammage_2012	Predicting exercise and eating behaviors from appearance evaluation and two types of investment	Canada	F/231	20.92 (2.62)	22.98	General university students	at the laboratory	Appearance Schemas Inventory-Revised (ASI-R) - motivational salience Subscale	Exercise Dependence Scale (EDS)	Cross-sectional	0.17
P14S3	Lamarche & Gammage_2012	Predicting exercise and eating behaviors from appearance evaluation and two types of investment	Canada	F/231	20.92 (2.62)	22.98	General university students	at the laboratory	Body-image Ideals Questionnaire (BIQ) - discrepancy subscale	Exercise Dependence Scale (EDS)	Cross-sectional	0.14

P15S1	LePage et al._2012	The effect of exercise absence on affect and body dissatisfaction as moderated by obligatory exercise beliefs and eating disordered beliefs and behaviors	USA	F/51	19.06 (3.10)	NA	Regular exercisers	10-day protocol	Body Shape Questionnaire (BSQ)	Obligatory Exercise Questionnaire (OEQ)	Longitudinal	0.54
P15S2	LePage et al._2012	The effect of exercise absence on affect and body dissatisfaction as moderated by obligatory exercise beliefs and eating disordered beliefs and behaviors	USA	F/76	19.08 (2.86)	NA	Regular exercisers	10-day protocol	Body Shape Questionnaire (BSQ)	Obligatory Exercise Questionnaire (OEQ)	Longitudinal	0.48
P16S1	Cook et al._2015	The influence of exercise identity and social physique anxiety on exercise dependence	USA	F/1766	36.98 (10.47)	23.78	Runners	online survey	Social Physique Anxiety Scale (SPAS)	Exercise Dependence Scale (EDS)	Cross-sectional	0.268
P17S1	Bell et al._2016	Is athletic really ideal? An examination of the mediating role of body dissatisfaction in predicting disordered eating and compulsive exercise	Australia	F/388	21.46 (4.51)	23	General university students	online survey	Body Image and Body Change Questionnaire - Body Image Concern subscale	Obligatory Exercise Questionnaire (OEQ)	Cross-sectional	0.05
P18S1	Brewster et al._2017	"Do You Even Lift, Bro?" Objectification, Minority Stress, and Body Image Concerns for Sexual Minority Men	USA	M/326	28.71 (18-62)	NA	General public	online survey	Objectified Body Consciousness Scale (OBCS) - body surveillance subscale	Compulsive Exercise Test (CET)	Cross-sectional	0.27
P18S2	Brewster et al._2017	"Do You Even Lift, Bro?" Objectification, Minority Stress, and Body Image Concerns for Sexual Minority Men	USA	M/326	28.71 (18-62)	NA	General public	online survey	Body Parts Satisfaction Scale for Men (BPSS-M)	Compulsive Exercise Test (CET)	Cross-sectional	0.16
P18S3	Brewster et al._2017	"Do You Even Lift, Bro?" Objectification, Minority Stress, and Body Image Concerns for Sexual Minority Men	USA	M/326	28.71 (18-62)	NA	General public	online survey	Drive for Muscularity Scale (DMS)	Compulsive Exercise Test (CET)	Cross-sectional	0.15
P19S1	Martin &	Personality traits and appearance-ideal	USA	F&M/5	19.37	24	General	online	Eating Pathology Symptoms	Commitment to	Cross-sec	0.09

	Racine_2017	internalization: Differential associations with body dissatisfaction and compulsive exercise		31	(2.2)		university students	survey system for course	Inventory (EPSI)-Body Dissatisfaction subscale	Exercise Scale (CES)	tional	
P20S1	Patterson & Goodson _2017	Using Social Network Analysis to Better Understand Compulsive Exercise Behavior among a Sample of Sorority Members	USA	F/206	NA	NA	General university students		single-factor Body Shape Questionnaire (BSQ)	Compulsive Exercise Test (CET)	Cross-sectional	0.553
P21S1	Ertl et al._2018	Running on empty: high self-esteem as a risk factor for exercise addiction	USA	F/322	19.89 (18-26)	NA	General university students	online	Objectified Body Consciousness Scale (OBC) - Body Shame Subscale	Exercise Addiction Inventory-Short Form (EAI)	Cross-sectional	0.37
P22S1	Uhlmann et al._2018	The fit beauty ideal: A healthy alternative to thinness or a wolf in sheep's clothing?	Australia	F/356	20.57 (20.57)	22.79	General university students	online	Body Image and Body Change Inventory - Body Image Concern subscale	Obligatory Exercise Questionnaire (OEQ)	Cross-sectional	0.03
P23S1	Liu et al._2019	Male weight trainers' body dissatisfaction and exercise dependence: Mediating role of muscularity drive	Taiwan, China	M/278	29.03 (7.83)	22.57	exercisers in fitness centers	meet	Muscular Figure Rating Scale (MFRS)	Exercise Dependence Scale-Revised (EDS-R)	Cross-sectional	0.22
P23S2	Liu et al._2019	Male weight trainers' body dissatisfaction and exercise dependence: Mediating role of muscularity drive	Taiwan, China	M/278	29.03 (7.83)	22.57	exercisers in fitness centers	meet	Drive for Muscularity Scale (DMS)	Exercise Dependence Scale-Revised (EDS-R)	Cross-sectional	0.54
P24S1	Donovan et	Strong is the New Skinny, but is it Ideal? A Test of the Tripartite Influence Model using a new Measure of	Australia	F/558	22.06 (7.41)	23.57	General university	online	Fit-Ideal Internalization Test (FIIT)	Compulsive Exercise Test (CET)	Cross-sectional	0.68

	al._2020	Fit-Ideal Internalization					students					
P24S2	Donovan et al._2020	Strong is the New Skinny, but is it Ideal? A Test of the Tripartite Influence Model using a new Measure of Fit-Ideal Internalization	Austr alia	F/558	22.06 (7.41)	23.57	General university students	online	Body-Image Ideals Questionnaire (BIQ)	Compulsive Exercise Test (CET)	Cross-sec tional	0.61
P25S1	Kelly et al._2020	Evaluating components of existing theories for loss of control eating in a sample of young racially/ethnically diverse men	USA	M/110 9	24.1 (3.6)	25.4	General public	online	Revised Male Body Image Attitudes Scale (MBAS-R) - body fat subscale	Exercise Dependence Scale (EDS)	Cross-sec tional	0.3
P25S2	Kelly et al._2020	Evaluating components of existing theories for loss of control eating in a sample of young racially/ethnically diverse men	USA	M/110 9	24.1 (3.6)	25.4	General public	online	Revised Male Body Image Attitudes Scale (MBAS-R) - muscularity subscale	Exercise Dependence Scale (EDS)	Cross-sec tional	0.22
P26S1	Dawson & Hammer_ 2020	No pain, no gains: Conformity to masculine norms, body dissatisfaction, and exercise dependence	USA	M/632	28.31 (18-59)	NA	General public	online	Male Body Attitudes Scale (MBAS) - Body Fat Dissatisfaction	Exercise Dependence Scale-Revised (EDS-R)	Cross-sec tional	0.07
P26S2	Dawson & Hammer_ 2020	No pain, no gains: Conformity to masculine norms, body dissatisfaction, and exercise dependence	USA	M/632	28.31 (18-59)	NA	General public	online	Male Body Attitudes Scale (MBAS) - Muscle Dissatisfaction	Exercise Dependence Scale-Revised (EDS-R)	Cross-sec tional	0.3
P27S1	Prochno w et al._2021	A social network approach to analyzing body dissatisfaction among sorority members using two network generators	USA	F/208	19.4 (1.2)	22.1	General university students	meet	single-factor Body Shape Questionnaire (BSQ)	Compulsive Exercise Test (CET)	Cross-sec tional	0.52
P28S1	Back et al._2021	Psychological risk factors for exercise dependence	Swed en	F&M/3 30	30.10 (18-60)	NA	exercisers in university exercise	Meetings	Multidimensional body-self relations questionnaire (MBSRQ) - appearance orientation subscale	Exercise dependence scale revised (EDS-R)	Cross-sec tional	0.19

							group					
P29S1	Gori et al._2021	Protective and Risk Factors in Exercise Addiction: A Series of Moderated Mediation Analyses	Italy	F&M/319	30.78 (11.98)	NA	Regular exercisers	online	Eating Disorder Inventory-3-Referral Form (EDI-3-RF) - body dissatisfaction subscale	Exercise Addiction Inventory (EAI)	Cross-sectional	0.172
P29S2	Gori et al._2021	Protective and Risk Factors in Exercise Addiction: A Series of Moderated Mediation Analyses	Italy	F&M/319	30.78 (11.98)	NA	Regular exercisers	online	Body Image Concern Inventory (BICI)	Exercise Addiction Inventory (EAI)	Cross-sectional	0.328
P30S1	Gori et al._2021	The Relationship between Alexithymia, Dysmorphic Concern, and Exercise Addiction: The Moderating Effect of Self-Esteem	Italy	F&M/288	28.35 (8.26)	NA	Regular exercisers	online	Body Image Concern Inventory (BICI) - dysmorphic symptoms subscale	Exercise Addiction Inventory (EAI)	Cross-sectional	0.305
P30S2	Gori et al._2021	The Relationship between Alexithymia, Dysmorphic Concern, and Exercise Addiction: The Moderating Effect of Self-Esteem	Italy	F&M/288	28.35 (8.26)	NA	Regular exercisers	online	Body Image Concern Inventory (BICI) - symptom interference subscale	Exercise Addiction Inventory (EAI)	Cross-sectional	0.298
P31S1	Bonfanti et al._2022	The Thin Ideal and Attitudes towards Appearance as Correlates of Exercise Addiction among Sporty People during the COVID-19 Pandemic	Italy	F&M/194	25.91 (6.32)	22.23	exercisers in fitness centers	online	Drive for Leanness Scale (DLS)	Exercise Addiction Inventory (EAI)	Cross-sectional	0.312
P32S1	Donova & Uhlmann _2022	Looking at me, looking at you: The mediating roles of body surveillance and social comparison in the relationship between fit ideal internalization and body dissatisfaction	Australia	F/448	18.83 (2.11)	23.05	General university students	online	Fit Ideal Internalization Test (FIIT)	Compulsive Exercise Test (CET)	Cross-sectional	0.67
P32S2	Donova & Uhlmann _2022	Looking at me, looking at you: The mediating roles of body surveillance and social comparison in the relationship between fit ideal internalization and body dissatisfaction	Australia	F/448	18.83 (2.11)	23.05	General university students	online	Objectified Body Consciousness Scale (OBCS) - body surveillance subscale	Compulsive Exercise Test (CET)	Cross-sectional	0.37
P32S3	Donova	Looking at me, looking at you: The mediating roles of	Austr	F/448	18.83	23.05	General	online	Body-Image Ideals	Compulsive	Cross-sec	0.49

	& Uhlmann _2022	body surveillance and social comparison in the relationship between fit ideal internalization and body dissatisfaction	alia		(2.11)		university students		Questionnaire (BIQ)	Exercise Test (CET)	tional	
P33S1	Novita et al._2022	Anxiety towards COVID-19, Fear of Negative Appearance, Healthy Lifestyle, and Their Relationship with Well-Being during the Pandemic A Cross-Cultural Study between Indonesia and Poland	Pola nd	F&M/7 09	32.39 (11.38)	NA	General university students	online	Fear of Negative Appearance Evaluation Scale (FNAES)	Obligatory Exercise Questionnaire (OEQ)	Cross-sec tional	0.197
P33S2	Novita et al._2022	Anxiety towards COVID-19, Fear of Negative Appearance, Healthy Lifestyle, and Their Relationship with Well-Being during the Pandemic a Cross-Cultural Study between Indonesia and Poland	Indo nesia	F&M/1 72	23.77 (7.73)	NA	General university students	online	Fear of Negative Appearance Evaluation Scale (FNAES)	Obligatory Exercise Questionnaire (OEQ)	Cross-sec tional	-0.15
P34S1	Mader et al._2023	Is (Disordered) Social Networking Sites Usage a Risk Factor for Dysfunctional Eating and Exercise Behavior?	Ger man y	F&M/1 22	25.85 (6.86)	NA	General public	online	Eating Disorder Examination Questionnaire (EDE-Q) - Shape concern scale	Exercise Dependence Scale-21 (EDS-21)	Cross-sec tional	-0.01
P34S2	Mader et al._2023	Is (Disordered) Social Networking Sites Usage a Risk Factor for Dysfunctional Eating and Exercise Behavior?	Ger man y	F&M/1 22	25.85 (6.86)	NA	General public	online	Eating Disorder Examination Questionnaire (EDE-Q) - Weight concern scale	Exercise Dependence Scale-21 (EDS-21)	Cross-sec tional	0.03
P35S1	Palermo & Rancourt _2023	Anxiety, body dissatisfaction, and exercise identity: Differentiating between adaptive and compulsive exercise	USA	F&M/4 46	20.10 (3.98)	NA	General university students	online	The Body Uneasiness Test-Body Image Concerns subscale	Exercise and Eating Disorder Questionnaire (EED) - Compulsive and Positive and Healthy Exercise subscales	Cross-sec tional	0.44

P36S1	Akbari et al._2024	The risk of exercise addiction mediates the relationship between social media use and mental health indices among young Iranians	Iran	F&M/745	26.19(7.42)	23.59	General public	online	Body Image Concern Inventory (BICI)	Exercise Addiction Inventory (EAI)	Cross-sectional	0.38
P37S1	Gori et al._2024	Family Functioning Styles and Exercise Addiction: Disengaged, Enmeshed, and Rigid Family Patterns Are Associated with Exercise Addiction	Italy	F&M/300	30.30(11.60)	NA	Regular exercisers	online	Body Image Concern Inventory (BICI)	Exercise Addiction Inventory (EAI)	Cross-sectional	0.32
P38S1	Soraci et al._2024	Psychometric properties of the Italian Tendency to Avoid Physical Activity and Sport Scale relationship to weight stigma and body esteem	Italy	F&M/235	35.17(10.83)	NA	General public	online	Body Esteem Scale	Exercise Addiction Inventory (EAI)	Cross-sectional	-0.04

Supplementary material F: **Forest plot**

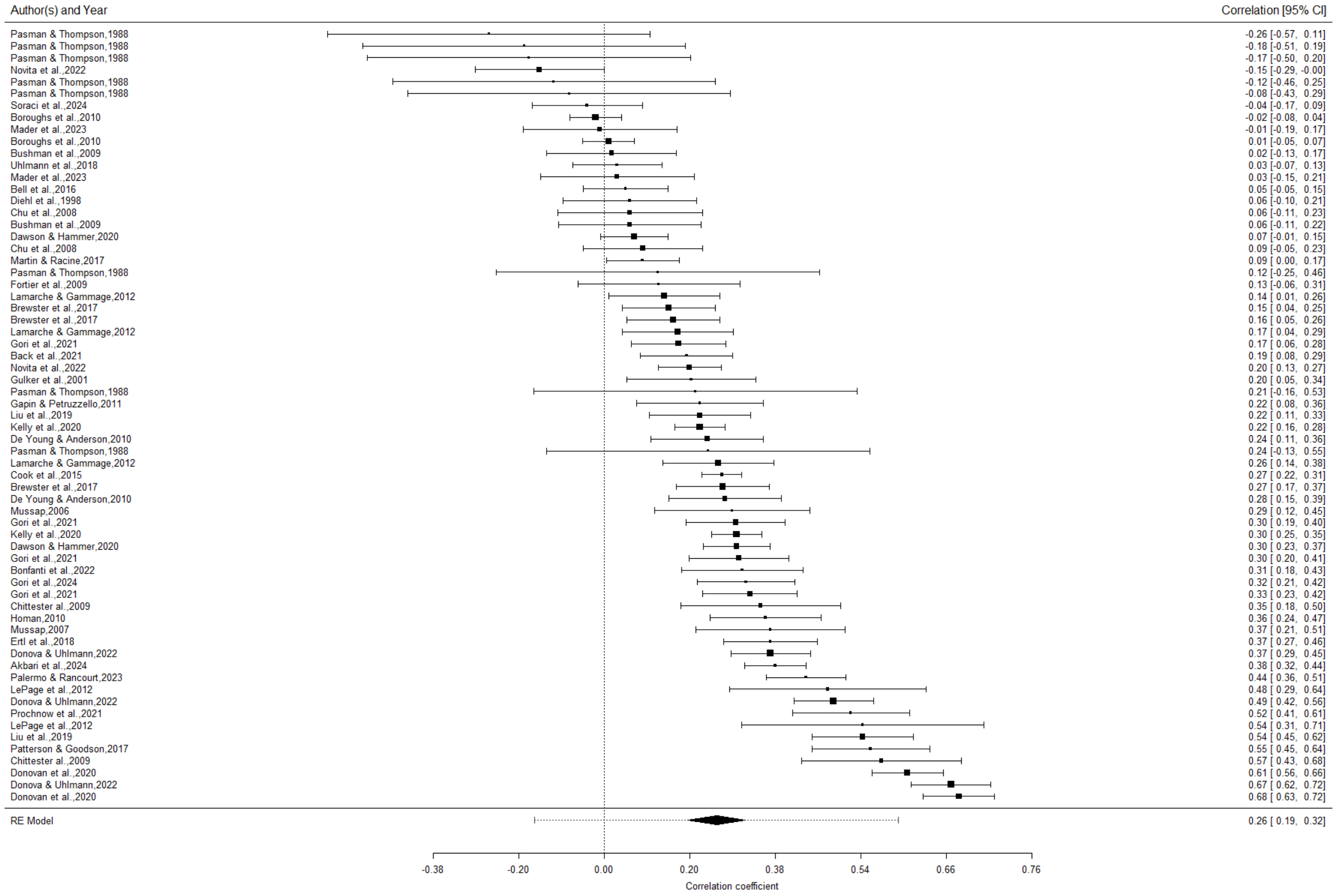

Supplementary Materials H

Result of univariable moderator analyses for the association between body image and exercise addiction.

Moderators	*s*	*k*	β_0	95% CI	Mean *r*	β_1	95% CI	$F_{(df1, df2)}$	*p*	%Var. Level 2	%Var. Level 3
**Type of body image measure**	38	65						$F_{(3, 61)} = 3.13$	0.032	29.26	63.25
Global and site-specific satisfaction		44	0.26	0.19, 0.33	0.25						
Affective measures		9	0.14	-0.02, 0.30	0.14	-0.12	-0.29, 0.06				
Behavioral measures		1	0.27	-0.05, 0.59	0.27	0.01	-0.31, 0.33				
Cognitive measures		11	0.41	0.29, 0.53	0.39	0.15	0.04, 0.27				
**Type of risk of exercise addiction measure**	38	65						$F_{(4, 60)} = 5.63$	<0.001	36.9	53.57
Obligatory exercise measure		28	0.16	0.07, 0.25	0.16						
Exercise dependence measures		15	0.26	0.14, 0.37	0.25	0.10	-0.05, 0.25				
Compulsive exercise measures		11	0.53	0.39, 0.66	0.48	0.37	0.21, 0.53				
Exercise addiction measures		9	0.28	0.15, 0.42	0.28	0.13	-0.03, 0.29				
Commitment to exercise measure		2	0.11	-0.16, 0.37	0.11	-0.05	-0.33, 0.23				
**Type of Participants**	38	65						$F_{(4, 60)} = 0.84$	0.506	24.5	69.37

General university students		31	0.29	0.19, 0.39	0.28						
General public		11	0.17	0.001, 0.35	0.17	-0.12	-0.31, 0.08				
Regular exercisers		9	0.30	0.12, 0.48	0.29	0.01	-0.19, 0.22				
Runners		6	0.32	0.09, 0.55	0.31	0.03	-0.22, 0.28				
Other populations		8	0.16	-0.05, 0.37	0.16	-0.13	-0.36, 0.10				
**Countries and regions**	38	65						$F_{(3, 61)} = 0.80$	0.501	27.03	66.47
North America		41	0.25	0.16, 0.34	0.24						
Australia		9	0.38	0.20, 0.55	0.36	0.13	-0.07, 0.32				
Europe		11	0.24	0.09, 0.39	0.24	-0.01	-0.18, 0.16				
Other countries and regions		4	0.18	-0.05, 0.40	0.18	-0.07	-0.31, 0.18				
**Study design**	38	65						$F_{(1, 63)} = 1.96$	0.167		
Cross-sectional		62	0.25	0.18, 0.32	0.25						
Longitudinal		3	0.47	0.17, 0.77	0.44	0.22	-0.09, 0.53				
**Gender**	38	65						$F_{(2, 62)} = 1.76$	0.181	29.16	63.93
Female		25	0.33	0.23, 0.43	0.32						
Male		18	0.27	0.14, 0.39	0.26	-0.07	-0.21, 0.08				
Mix-gender		22	0.20	0.10, 0.30	0.20	-0.13	-0.28, 0.01				
**Q-SSP Score**	38	65	0.26	0.19, 0.33	0.26	0.00	-0.01, 0.01	$F_{(1, 63)} = 0.01$	0.913	29.42	63.51
**Year of publication**	38	65	0.24	0.17, 0.31	0.24	0.01	-0.00, 0.02	$F_{(1, 63)} = 3.67$	0.06	29.42	63.51
**Age**	33	51	0.28	0.21, 0.35	0.28	-0.01	-0.02, 0.01	$F_{(1, 49)} = 1.25$	0.27	35.86	56.98
**BMI**	17	25	0.33	0.22, 0.45	0.32	0.02	-0.10, 0.13	$F_{(1, 23)} = 0.07$	0.791	38.82	55.63

Note: s = Number of studies; k = Number of effect sizes; β_0 = Intercept/mean effect size (z); CI = Confidence interval; Mean r = mean effect size r; β_1 = Estimated regression coefficient; $F_{(df1, df2)}$ = Omnibus test; p = the significance test of moderating effect test; %Var = percentage of variance explained.
